# Early Chronic Memantine Treatment-Induced Transcriptomic Changes in Wild-Type and *Shank2*-Mutant Mice

**DOI:** 10.3389/fnmol.2021.712576

**Published:** 2021-09-14

**Authors:** Ye-Eun Yoo, Seungjoon Lee, Woohyun Kim, Hyosang Kim, Changuk Chung, Seungmin Ha, Jinsu Park, Yeonseung Chung, Hyojin Kang, Eunjoon Kim

**Affiliations:** ^1^Department of Biological Sciences, Korea Advanced Institute of Science and Technology, Daejeon, South Korea; ^2^Center for Synaptic Brain Dysfunctions, Institute for Basic Science, Daejeon, South Korea; ^3^Department of Mathematical Sciences, Korea Advanced Institute of Science and Technology, Daejeon, South Korea; ^4^Division of National Supercomputing, Korea Institute of Science and Technology Information, Daejeon, South Korea

**Keywords:** autism spectrum disorders, *Shank2*, NMDA receptor, memantine, RNA-Seq, synapse, ribosome, mitochondria

## Abstract

Shank2 is an excitatory postsynaptic scaffolding protein strongly implicated in autism spectrum disorders (ASDs). *Shank2*-mutant mice with a homozygous deletion of exons 6 and 7 (*Shank2*-KO mice) show decreased NMDA receptor (NMDAR) function and autistic-like behaviors at juvenile [∼postnatal day (P21)] and adult (>P56) stages that are rescued by NMDAR activation. However, at ∼P14, these mice show the opposite change – increased NMDAR function; moreover, suppression of NMDAR activity with early, chronic memantine treatment during P7–21 prevents NMDAR hypofunction and autistic-like behaviors at later (∼P21 and >P56) stages. To better understand the mechanisms underlying this rescue, we performed RNA-Seq gene-set enrichment analysis of forebrain transcriptomes from wild-type (WT) and *Shank2*-KO juvenile (P25) mice treated early and chronically (P7–21) with vehicle or memantine. Vehicle-treated *Shank2*-KO mice showed upregulation of synapse-related genes and downregulation of ribosome- and mitochondria-related genes compared with vehicle-treated WT mice. They also showed a transcriptomic pattern largely opposite that observed in ASD (reverse-ASD pattern), based on ASD-related/risk genes and cell-type–specific genes. In memantine-treated *Shank2*-KO mice, chromatin-related genes were upregulated; mitochondria, extracellular matrix (ECM), and actin-related genes were downregulated; and the reverse-ASD pattern was weakened compared with that in vehicle-treated *Shank2*-KO mice. In WT mice, memantine treatment, which does not alter NMDAR function, upregulated synaptic genes and downregulated ECM genes; memantine-treated WT mice also exhibited a reverse-ASD pattern. Therefore, early chronic treatment of *Shank2*-KO mice with memantine alters expression of chromatin, mitochondria, ECM, actin, and ASD-related genes.

## Introduction

Shank2 (also known as ProSAP1) is an abundant excitatory postsynaptic scaffolding protein [(Du et al., 1998; Boeckers et al., 1999; Lim et al., 1999; Naisbitt et al., 1999); reviewed in Boeckers et al. (2002); Grabrucker et al. (2011), Mossa et al. (2017); Sala et al. (2015), and Sheng and Kim (2000, 2011)] implicated in autism spectrum disorders (ASD), intellectual disability, developmental delay, and schizophrenia (Berkel et al., 2010, 2011; Pinto et al., 2010; Leblond et al., 2012, 2014; Rauch et al., 2012; Sanders et al., 2012; Chilian et al., 2013; Liu et al., 2013; Guilmatre et al., 2014; Costas, 2015; Peykov et al., 2015a,b; Homann et al., 2016; Mossa et al., 2017; Yuen et al., 2017; Bai et al., 2018; Lu et al., 2018; Satterstrom et al., 2020; Wang et al., 2020).

*Shank2*-mutant mice with a homozygous deletion of exons 6 and 7, hereafter termed *Shank2*-KO mice, show decreased NMDA receptor (NMDAR) currents, through mechanisms equally affecting GluN2A- and GluN2B-containing NMDARs, although additional details remain to be determined (Won et al., 2012). These mice also show social deficits at juvenile [∼postnatal (P25)] and adult (∼P56) stages that are rescued by acute treatment with the NMDAR agonist D-cycloserine (Won et al., 2012). Intriguingly, the same *Shank2*-KO mice show an opposite change in NMDAR function – NMDAR hyperactivity – at ∼P14 that is thought to cause NMDAR hypoactivity at ∼P21 and later stages, as supported by the fact that early, chronic (P7–21) treatment with memantine, a low-affinity uncompetitive antagonist of extrasynaptic NMDARs (Lipton, 2006) with beneficial effects on various brain disorders, including Alzheimer’s disease (Matsunaga et al., 2015; Folch et al., 2018), normalizes NMDAR function at juvenile (∼P21) and adult (>P56) stages (Chung et al., 2019; Verpelli et al., 2019). These results suggest that early NMDAR hyperactivity at P14 induces NMDAR hypoactivity at P21 that persists into adulthood, and that early correction of NMDAR hyperactivity may prevent the compensatory changes that lead to late-stage NMDAR hypoactivity. However, how early, chronic memantine treatment prevents synaptic and behavioral deficits in juvenile and adult *Shank2*-KO mice remains unclear. In addition, it is unclear whether early memantine treatment in *Shank2*-KO mice restores wild-type (WT) mouse-like phenotypes by reversing molecular and cellular changes or induces some unique changes that account for the phenotypic reversal.

To address these questions, we treated WT and *Shank2*-KO mice early and chronically (P7–21) with vehicle or memantine and performed RNA-Seq analyses of the forebrain, with RNA preparation at P25 for a few days of stabilization after cessation of drug/vehicle treatment. We chose memantine over other NMDAR modulators such as D-cycloserine (an NMDAR agonist) and clioquinol (a zinc chelator), which also rescue *Shank2*-KO mouse phenotypes upon acute treatments (Won et al., 2012; Lee et al., 2015). It was because, unlike acute D-cycloserine and clioquinol treatments with direct NMDAR modulation, chronic memantine treatment would likely to exert not only NMDAR modulation but also additional effects downstream of NMDAR modulation such as regulation of gene expression.

The RNA-Seq analyses indicate that early memantine treatment of *Shank2*-KO mice induced upregulation of chromatin-related genes and downregulation of mitochondria/extracellular matrix (ECM)-related genes. It also induced transcriptomic changes, altering ASD-related/risk genes in a way that weakened the strong reverse-ASD nature of the transcriptome compared with that in vehicle-treated *Shank2*-KO mice. In WT mice, where memantine does not change NMDAR function, memantine induced reverse-ASD transcriptomic changes that involved upregulation of synaptic genes and downregulation of ECM genes.

## Materials and Methods

### Animals

*Shank2* mouse line lacking exons 6 + 7 has been reported previously (Won et al., 2012) and deposited at Jackson Laboratory (B6N.129S4-*Shank2^TM 1*Mgle*^*/CsbdJ; Jackson 033667). Mice were fed *ad libitum* under a 12-h dark/light cycle. All mice were housed and bred at the mouse facility of Korea Advanced Institute of Science and Technology (KAIST) and maintained according to the Animal Research Requirements of KAIST. The animal study was approved by the Committee of Animal Research at KAIST (KA2020-99).

### Drug Treatment and Sample Preparation for RNA-Seq

Three mice per group (four groups from vehicle or memantine-treated WT and homozygous *Shank2*-knockout/KO mice) were used without pooling of the brains for RNA-Seq analyses. Early and chronic memantine was administered as previously described (Chung et al., 2019). Briefly, memantine hydrochloride (Sigma, M9292; 20 mg/kg; in 0.1% saccharin) was orally administered twice-a-day for 2 weeks (P7–21). At P25, mice were anesthetized with isoflurane, and the forebrain region, remained after the removal of the cerebellum and brain stem structures beneath the cerebellum, were soaked in RNAlater solution (Ambion), chopped into small pieces, and kept at −70°C. Poly-A mRNAs were purified using poly−T oligo−attached magnetic beads, followed by RNA fragmentation using divalent cations under elevated temperature. RNA concentrations were calculated using Quant-IT RiboGreen (Invitrogen, R11490), and RNA integrity was determined using TapeStation RNA screen tape (Agilent Technologies), after which only high-quality RNAs (RIN > 7.0) were used for cDNA library construction using Illumina TruSeq mRNA Sample Prep kit (Illumina). Indexed libraries were submitted to an Illumina NovaSeq (Illumina), and paired-end (2 × 100 bp) sequencing was performed by Macrogen Inc.

### RNA-Seq Analysis

Transcript abundance was estimated with Salmon (v1.1.0) (Patro et al., 2017) in Quasi-mapping-based mode onto the *Mus musculus* genome (GRCm38) with GC bias correction (–gcBias). Quantified gene-level abundance data was imported to R (v.3.5.3) with the tximport (Soneson et al., 2015) package and differential gene expression analysis was carried out using R/Bioconductor DEseq2 (v1.30.1) (Love et al., 2014). Principal component analysis (PCA) was performed for the regularized log transform (rlog) of the normalized counts using plotPCA (with default parameter) tools implemented in DEseq2. Normalized read counts were computed by dividing the raw read counts by size factors and fitted to a negative binomial distribution. The *p*-values were adjusted for multiple testing with the Benjamini–Hochberg correction. Genes with an adjusted *p*-value of less than 0.05 were considered as differentially expressed. Volcano plots were generated using the R ggplot2 (v.3.1.1) package. The Gene Ontology (GO) enrichment analyses were performed using DAVID software (version 6.8) (Huang da et al., 2009). Mouse gene names were converted to human homologs using the Mouse Genome Informatics (MGI) database.^1^ Gene Set Enrichment Analysis (GSEA)^2^ (Subramanian et al., 2005) was used to determine whether *a priori*-defined gene sets would show statistically significant differences in expression between WT and *Shank2*-mutant mice. Enrichment analysis was performed using GSEAPreranked^®^ (gsea-3.0.jar) module on gene set collections downloaded from Molecular Signature Database (MSigDB) v7.0.^3^ GSEAPreranked was applied using the list of all genes expressed, ranked by the fold change and multiplied by the inverse of the *p*-value with recommended default settings (1,000 permutations and a classic scoring scheme). The false discovery rate (FDR) was estimated to control the false positive finding of a given normalized enrichment score (NES) by comparing the tails of the observed and null distributions derived from 1,000 gene set permutations. The gene sets with an FDR of less than 0.05 were considered as significantly enriched. Integration and visualization of the GSEA results were performed using the EnrichmentMap Cytoscape App (version 3.8.1) (Merico et al., 2010; Isserlin et al., 2014).

### RT-qPCR

Top individual genes contributing most strongly to the gene-set enrichments were validated using RT-qPCR using M-MLV cDNA synthesis kit (Enzynomics, EZ006). qPCR was performed using THUNDERBIRD^TM^ SYBR^®^ qPCR mix (TOYOBO, QPS-201), CFX96^TM^ Real-Time system (BIO-RAD). Genes and primer-set information are shown below:

**Table:** 

HTR2A	Forward	TAGCCGCTTCAACTCCAGAA
	Reverse	AAGACTGGGATTGGCATGGA
MUC1	Forward	GCATCAAGTTCAGGTCAGGC
	Reverse	GACTTCACGTCAGAGGCACTA
SF3B1	Forward	CGAACAGATCGAGGTGGAGA
	Reverse	GGAGTCAGAACAGGAGTGCT
GAPDH	Forward	CATCACTGCCACCCAGAAGACTG
	Reverse	ATGCCAGTGAGCTTCCCGTTCAG

### Statistical Power Analysis

We performed a power analysis to evaluate if the sample size of 3 per group leads to enough statistical power to detect the truly differentially expressed genes (DEGs) among vehicle/memantine-treated WT and Shank2-KO mice. For each of the 6 comparisons (Veh-K/W, Mem-K/W, KO-M/V, K-M/W-V, WT-M/V, WT-M/KO-V), we simulated the data for normalized read counts from the negative binomial models where the parameters were set based on the results of the real data analysis. The statistical power was calculated as TP/(TP + FN) where TP and FN refer to true positive and false negative, respectively, as described previously (Ching et al., 2014). We performed 100 simulations and varied the sample size as 3, 6, and 9.

## Results

### Analysis of Differentially Expressed Genes in Vehicle- or Memantine-Treated WT and *Shank2*-KO Mice

**FIGURE 1:**
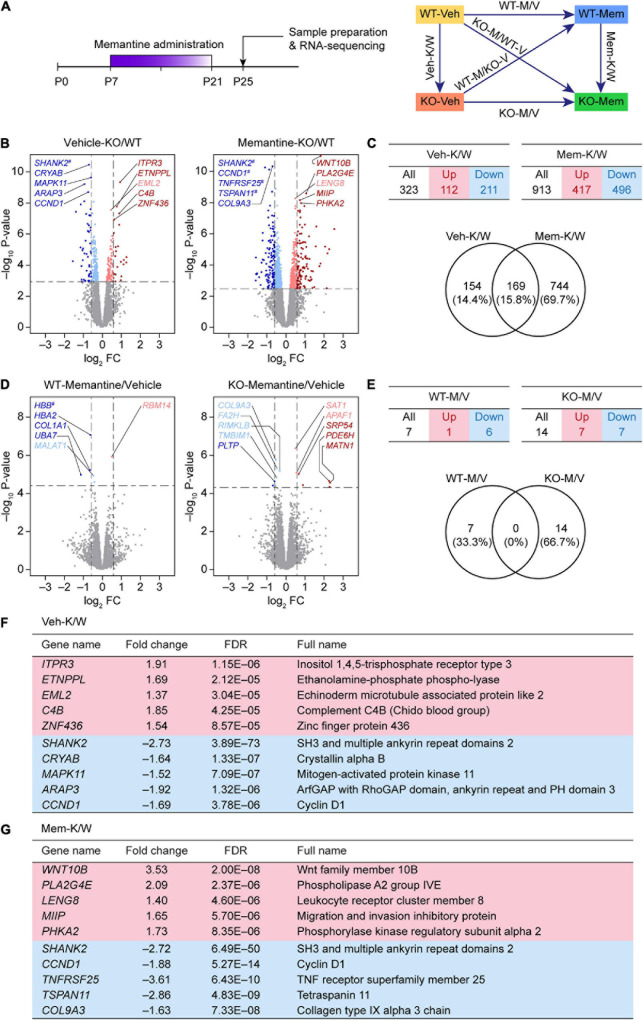
Differentially expressed gene analyses based on RNA-Seq results from WT-V, WT-M, *Shank2*-KO-V, and *Shank2*-KO-M mice. **(A)** Schematic depiction of RNA-Seq analyses employing vehicle (Veh)- or memantine (Mem)-treated WT and *Shank2*-KO mice, and DEG and GSEA analyses comparing the effects of memantine treatment between WT and KO mice, or differences between WT and KO mice under vehicle- and memantine-treatment conditions. **(B,C)** Volcano plot and summary table for DEGs from Veh-KO/WT (Veh-K/W) and memantine-KO/WT (Mem-K/W) transcripts, and Venn diagram showing the overlap between DEGs from Veh-K/W and Mem-K/W transcripts. (*n* = 3 mice for WT-V, WT-M, KO-V, and KO-M; dotted lines indicate adjusted *p*-value < 0.05 or |FC| > 1.5; DEGs were defined by adjusted *p*-values but not fold changes). **(D,E)** Volcano plot and summary table for DEGs from WT-memantine/vehicle (WT-M/V) and KO-memantine/vehicle (KO-M/V) transcripts, and Venn diagram showing the overlap between DEGs from WT-M/V and KO-M/V transcripts. Gene names marked with a hashtag without linkage to the indicated dots indicate those that have fold-change values that exceed the range of the axis. (*n* = 3 mice for WT-V, WT-M, KO-V, and KO-M; dotted lines indicate adjusted *p*-value < 0.05 or |FC| > 1.5; DEGs were defined by adjusted *p*-values but not fold changes). **(F,G)** Lists of top five up- and downregulated genes in Veh-K/W and Mem-K/W transcripts (see Supplementary Table 2 for further details).

To explore molecular and cellular mechanisms that underlie the rescue of phenotypes in *Shank2*-KO mice by early memantine treatment, we performed RNA-Seq analysis of transcriptomes in the forebrain region (excluding the cerebellum and underlying brain stem region) in WT and *Shank2*-KO mice (homozygous deletion of exons 6 and 7) at P25 treated early and chronically (P7–21) with vehicle or memantine (three mice per group) using a dose (20 mg/kg, oral, twice-a-day) identical to that previously rescued synaptic and behavioral phenotypes in *Shank2*-KO mice (Chung et al., 2019; Figure 1A, Supplementary Table 1, and Supplementary Figure 1A). The twice-a-day treatment considered the short elimination half-life (<4 h) of memantine in rodents (Beconi et al., 2011). We did not analyze specific brains of *Shank2*-KO mice because various ASD-related behavioral deficits responsive to memantine in *Shank2*-KO mice (i.e., social, repetitive, hyperactivity) could not be readily associated with particular brain regions, although a previous study has reported brain region-specific regulations of NMDARs in another *Shank2*-KO mouse line (deletion of exon 7) (Schmeisser et al., 2012).

In analyzing RNA-Seq results, we sought to compare transcriptomes from all six possible experimental combinations for the indicated reasons by examining transcripts from: (1) vehicle-treated WT and KO mice (termed Veh-K/W transcripts), to determine baseline differences between WT and KO mice; (2) memantine-treated WT and KO mice (Mem-K/W transcripts), to determine how memantine treatment alters baseline differences between WT and KO mice; (3) vehicle- and memantine-treated KO mice (KO-M/V transcripts), to determine how memantine treatment alters transcriptomic patterns in KO mice; (4) vehicle-treated WT and memantine-treated KO mice (K-M/W-V transcripts), to determine whether memantine treatment in KO mice restores transcriptomic patterns to a WT-like pattern; (5) vehicle- and memantine-treated WT mice (WT-M/V transcripts), to determine how memantine treatment in WT mice changes transcriptomic patterns; and (6) memantine-treated WT and vehicle-treated KO mice (WT-M/KO-V transcripts), to determine whether memantine-treated WT mice show transcriptomic changes similar to those induced by *Shank2* KO.

We first attempted an analysis of DEGs between vehicle-treated WT and KO mice (Veh-K/W transcripts), identifying a total of 323 DEGs (112 upregulated and 211 downregulated) that differed between WT and KO mice under baseline conditions (Figures 1B,C and Supplementary Table 2).

An analysis of transcripts from memantine-treated WT and KO mice (Mem-K/W transcripts) revealed 913 DEGs (417 upregulated and 496 downregulated) (Figures 1B,C), an increase of approximately threefold compared with that in vehicle-treated WT and KO mice. About 20% of Mem-K/W DEGs (169 DEGs) overlapped with Veh-K/W DEGs, indicating that transcriptomic changes in Mem-K/W transcripts are largely unique; this compares with an approximate 50% overlap within Veh-K/W DEGs (Figure 1C). The overlapped 169 DEGs showed identical directions (up or down) of transcriptional changes (100%) (Supplementary Table 2).

In contrast to these changes, there was a relatively small number of DEGs (*n* = 14; seven upregulated and seven downregulated) between transcripts from memantine- and vehicle-treated *Shank2*-KO mice (KO-M/V transcripts) (Figures 1D,E and Supplementary Table 2). Similarly, transcripts from memantine- and vehicle-treated WT mice (WT-M/V transcripts) showed small numbers of DEGs (*n* = 7; one upregulated and six downregulated), with no overlap between KO-M/V and WT-M/V DEGs (Figures 1D,E and Supplementary Table 2). These results suggest that early, chronic memantine treatment broadens the difference between WT and KO transcriptomes, whereas memantine treatment has minimal impacts on transcriptomes within WT or KO mice, at least in our DEG analyses.

Top five upregulated genes in Veh-K/W transcripts included *Itpr3* (inositol 1,4,5-trisphosphate receptor type 3), *Etnppl* (ethanolamine-phosphate phospho-lyase), *Eml2* (echinoderm microtubule associated protein like 2), *C4B* (complement C4B), and *Znf436* (zinc finger protein 436) (Figure 1F). Top five downregulated genes in Veh-K/W transcripts included *Shank2* (SH3 and multiple ankyrin repeat domains 2), *Cryab* (crystallin alpha B), *Mapk11* (mitogen-activated protein kinase 11), *Arap3* (ArfGAP with RhoGAP domain, ankyrin repeat, and PH domain 3), and *Ccnd1* (cyclin D1). Top five upregulated genes in Mem-K/W transcripts included *Wnt10b* (Wnt family member 10B), *Pla2g4e* (phospholipase A2 group IVE), *Leng8* (leukocyte receptor cluster member 8), *Miip* (migration and invasion inhibitory protein), and *Phka2* (phosphorylase kinase regulatory subunit alpha 2) (Figure 1G). Top five downregulated genes in Mem-K/W transcripts included *Shank2*, *Ccnd1* (cyclin D1), *Tnfrsf25* (TNF receptor superfamily member 25), and *Tspan11* (tetraspanin 11), *Col9a3* (collagen type IX alpha 3 chain).

Lastly, we performed a power analysis to evaluate if the sample size of 3 per group leads to enough statistical power. In all of the six comparisons, the power was above 0.64 when the sample size per group was 3 (Supplementary Figure 1B). For the comparison where the number of DEGs was small, the power was higher (i.e., above 0.75 for WT-M/V and KO-M/V transcript groups). These results indicate that the sample size of 3 per group provides reasonably high statistical power to detect DEGs in our study.

### Functional Annotations of Veh-K/W and Mem-K/W DEGs

We next attempted functional analyses of Veh-K/W and Mem-K/W DEGs using the GO analysis tool, Enrichr (Chen et al., 2013; Kuleshov et al., 2016). WT-M/V and KO-M/V DEGs were not used in the Enrichr analysis because their numbers were small. We also combined up- and downregulated DEGs to increase the power of functional annotations.

**FIGURE 2:**
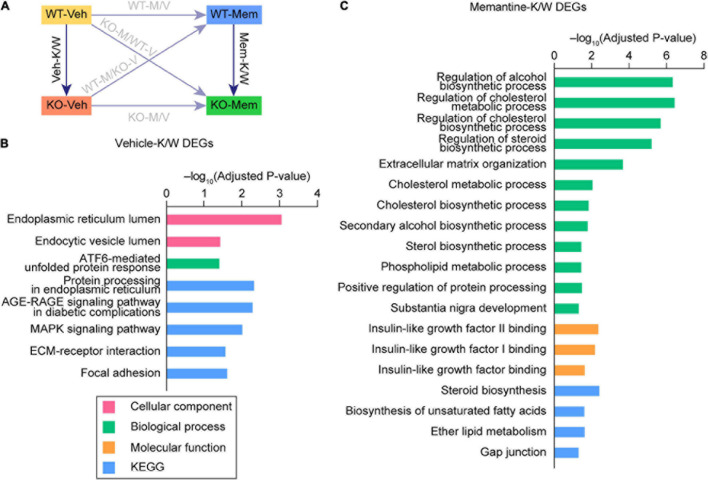
Enrichr Gene Ontology (GO) analyses of DEGs for Veh-KO/WT and Mem-KO/WT transcripts. **(A)** Schematic depiction highlighting the differences between WT and KO mice under vehicle- and memantine-treatment conditions. **(B,C)** Enrichr GO analysis of DEGs (defined by adjusted *p*-values < 0.05) for Veh-KO/WT (Veh-K/W; **B**) and memantine-KO/WT (Mem-K/W; **C**) transcripts in cellular component (CC), biological process (BP), molecular function (MF), and KEGG domains (adjusted *p*-value < 0.05). DEGs for WT-M/V and KO-M/V were not subjected to Enrichr analysis because of their small numbers.

Veh-K/W DEGs (baseline WT–KO difference) were annotated to GO terms that included endoplasmic reticulum, unfolded protein response, and MAP kinase in cellular component (CC), biological process (BP), and Kyoto Encyclopedia of Genes and Genomes (KEGG) domains (Figures 2A,B and Supplementary Table 3). Mem-K/W DEGs were annotated to GO terms associated with steroid/lipid metabolism, insulin-like growth factor, and ECM, and gap junction in BP, molecular function (MF), and KEGG domains (Figure 2C and Supplementary Table 3). These results suggest that vehicle-treated WT and KO mice and memantine-treated WT and KO mice show largely distinct transcriptomic changes, as analyzed by GO functional annotations.

### Gene Set Enrichment Analysis of Veh-K/W Transcripts

**FIGURE 3:**
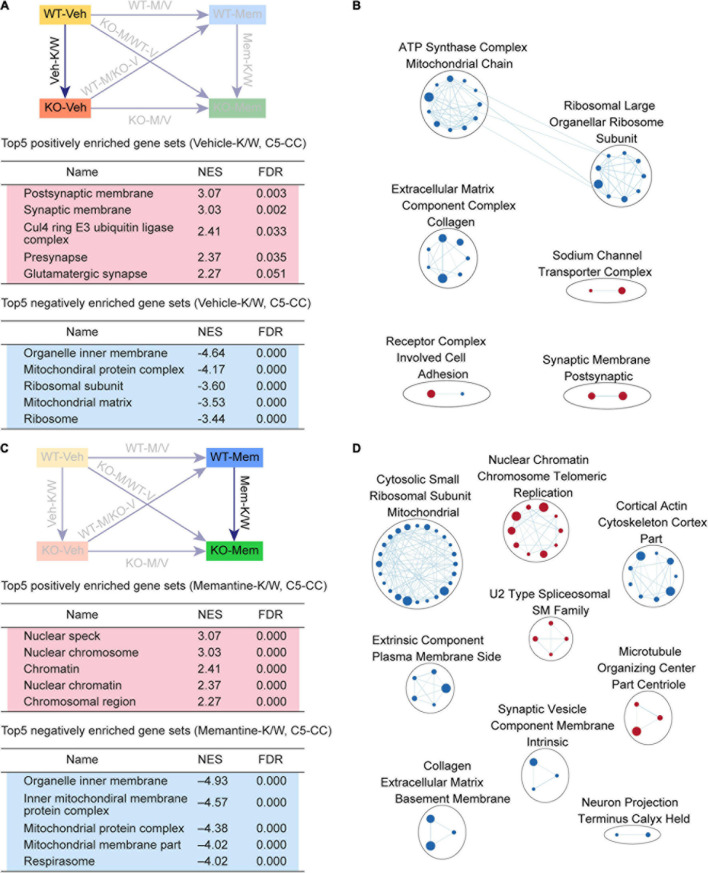
Gene Set Enrichment Analysis of Veh-K/W and Mem-K/W transcripts for biological functions. **(A,B)** GSEA results for Veh-K/W transcripts showing a list of the top five positively (red) and negatively (blue) enriched gene sets **(A)** and their integrated visualization, generated using the Cytoscape App, EnrichmentMap **(B)**. Only the results for C5-CC are shown here; those for C5-BP and C5-MF are shown in Supplementary Figure 1. Note that only the top five gene sets are shown in the main figure tables for simplicity (see the full lists in Supplementary Tables). All GSEA results were visualized using EnrichmentMap. Each circle in a cluster indicates significantly (FDR < 0.05) enriched individual gene sets with the size and color (red/blue) of the circles indicating gene-set size and positive/negative enrichment based on NES scores, respectively. [*n* = 3 mice (WT-Veh, KO-Veh), FDR < 0.05]. **(C,D)** GSEA results for Mem-K/W transcripts showing a list of the top five positively (red) and negatively (blue) enriched gene sets **(C)** and their integrated visualization generated using the EnrichmentMap Cytoscape App **(D)**. See Supplementary Figure 2 for GSEA C5-BP/MF results. [*n* = 3 mice (WT-Mem, KO-Mem), FDR < 0.05].

We next attempted GSEA, which uses the entire list of genes with transcriptional changes ranked *p*-values or fold changes, rather than a small portion of genes with an artificial cutoff, to identify the changes in biological functions that are driven by large number of genes with moderate but coordinate changes^4^ (Mootha et al., 2003; Subramanian et al., 2005). Veh-K/W transcripts (baseline WT–KO difference), ranked by *p*-values, were enriched for positively enriched (or upregulated genes were more strongly enriched) for synapse-related gene sets in CC, BP, and MF domains of the C5 gene sets (GO gene sets; 10,185 gene sets as of now) (Figure 3A, Supplementary Figure 2, and Supplementary Table 4). Integration and visualization of the enriched gene sets using EnrichmentMap Cytoscape App (Merico et al., 2010; Isserlin et al., 2014) further highlighted synapse and receptor/channel-related functions (Figure 3B).

Veh-K/W transcripts were negatively enriched for ribosome- and mitochondria-related gene sets (Figure 3A). In addition, an EnrichmentMap analysis highlighted clusters of gene sets associated with ribosome-, mitochondria-, and ECM-related functions (Figure 3B). These results suggest that there is a clear baseline transcriptomic difference between vehicle-treated WT and *Shank2*-KO mice, including upregulated synapse-related genes and downregulated ribosome/mitochondria/ECM-related genes.

### GSEA of Mem-K/W Transcripts

To determine if early memantine treatment alters the baseline transcriptomic difference between vehicle-treated WT and *Shank2*-KO mice, we compared memantine-treated WT and *Shank2*-KO transcripts (Mem-K/W transcripts). Mem-K/W transcripts were positively enriched for gene sets associated with chromatin functions, which were integrated into EnrichmentMap clusters of chromatin- and RNA splicing-related gene sets (Figures 3C,D, Supplementary Figure 3, and Supplementary Table 4), a pattern distinct from the upregulated synapse-related genes in Veh-K/W transcripts.

Mem-K/W transcript were also negatively enriched for gene sets associated with ribosome and mitochondria functions, with EnrichmentMap analyses showing clustering of ribosome-, mitochondria-related gene sets (Figures 3C,D), a pattern similar to that observed in Veh-K/W transcripts. These results suggest that early memantine treatment upregulates chromatin- and RNA-splicing-related genes and downregulates ribosome/mitochondria-related genes in the context of differences between WT and KO. The positive enrichment for synapse-related function, observed in Veh-K/W, was not present in Mem-K/W transcripts.

### GSEA of KO-M/V Transcripts

**FIGURE 4:**
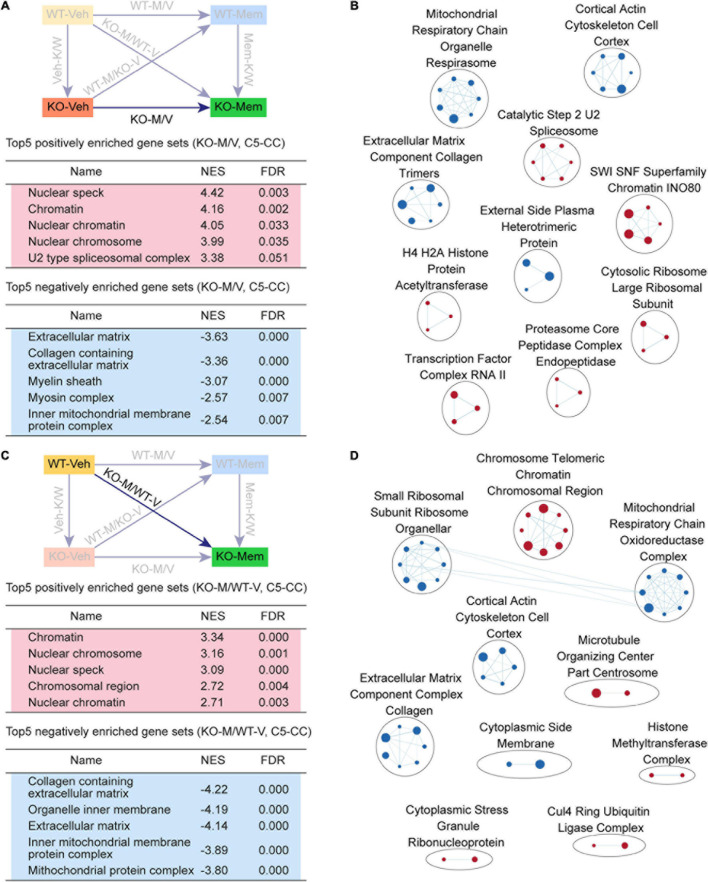
Gene Set Enrichment Analysis of KO-M/V and KO-M/WT-V transcripts for biological functions. **(A,B)** GSEA results for KO-M/V transcripts showing a list of the top five positively (red) and negatively (blue) enriched gene sets in the C5-CC domain **(A)** and their integrated visualization generated using EnrichmentMap **(B)**. See Supplementary Figure 3 for GSEA C5-BP/MF results. [*n* = 3 mice (KO-Veh, KO-Mem), FDR < 0.05]. **(C,D)** GSEA results for KO-M/WT-V transcripts showing a list of the top five positively (red) and negatively (blue) enriched gene sets in the C5-CC domain **(C)** and their integrated visualization generated using EnrichmentMap **(D)**. See Supplementary Figure 4 for GSEA C5-BP/MF results. [*n* = 3 mice (WT-Veh, KO-Mem), FDR < 0.05].

We next sought to determine whether early memantine treatment changes transcriptomic patterns in *Shank2*-KO mice. We found that KO-M/V transcripts were positively enriched for chromatin- and RNA splicing-related functions (Figure 4A, Supplementary Figure 4, and Supplementary Table 5), similar to the upregulation of chromatin and RNA splicing functions among Mem-K/W transcripts. Chromatin and RNA splicing functions were further highlighted by EnrichmentMap analyses (Figure 4B). The positive enrichment for synapse-related function, observed in Veh-K/W, was not present in KO-M/V transcripts, similar to the pattern in Mem-K/W transcripts.

KO-M/V transcripts were negatively enriched for mitochondria- and ECM-related functions (Figure 4A), exhibiting EnrichmentMap patterns for mitochondria and ECM functions (Figure 4B). These results suggest that early memantine treatment of *Shank2*-KO mice upregulates chromatin- and splicing-related genes and downregulates mitochondria- and ECM-related genes, differences that are largely similar to the transcriptomic differences observed among Mem-K/W transcripts and are likely involved in memantine-dependent phenotypic rescue in *Shank2*-KO mice.

### GSEA of KO-M/WT-V Transcripts

We next compared the transcriptomic patterns of early memantine-treated *Shank2*-KO and vehicle-treated WT mice (KO-M/WT-V transcripts) to determine whether early memantine treatment normalizes phenotypic deficits in *Shank2*-KO mice by reversing transcriptomic patterns – restoring them to a pattern similar to that of the WT transcriptome – or rescues phenotypes by inducing distinct transcriptomic changes.

KO-M/WT-V transcripts were positively enriched for chromatin-related gene sets and negatively enriched for ribosome/mitochondria/ECM-related gene sets, as further supported by EnrichmentMap integration (Figure 4C,D, Supplementary Figure 5, and Supplementary Table 5). These results suggest that the KO-Mem transcriptome is distinct from the WT-Veh transcriptome and that early memantine treatment does not reverse the transcriptome of *Shank2*-KO mice to a pattern similar to that of the WT-Veh transcriptome.

### GSEA of WT-M/V and WT-M/KO-V Transcripts

**FIGURE 5:**
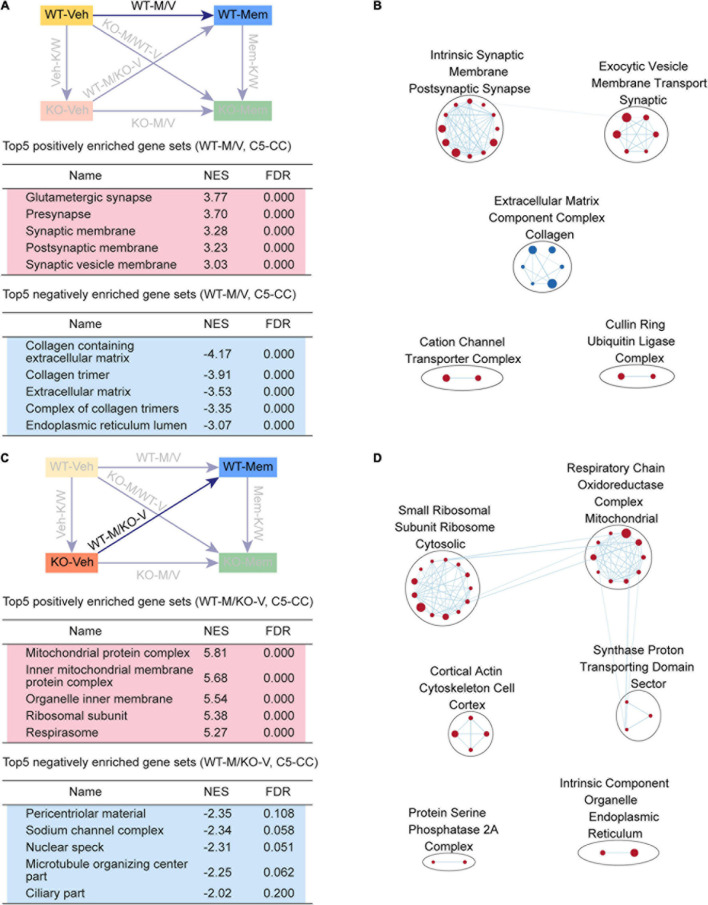
Gene Set Enrichment Analysis of WT-M/V and WT-M/KO-V transcripts for biological functions. **(A,B)** GSEA results for WT-M/V transcripts showing a list of the top five positively (red) and negatively (blue) enriched gene sets in the C5-CC domain **(A)** and their integrated visualization generated using EnrichmentMap **(B)**. See Supplementary Figure 5 for GSEA C5-BP/MF results. [*n* = 3 mice (WT-Veh, WT-Mem), FDR < 0.05]. **(C,D)** GSEA results for WT-M/KO-V transcripts showing a list of the top five positively (red) and negatively (blue) enriched gene sets in the C5-CC domain **(C)** and their integrated visualization generated using EnrichmentMap **(D)**. See Supplementary Figure 6 for GSEA C5-BP/MF results. [*n* = 3 ice (WT-Mem, KO-Veh), FDR < 0.05].

We compared vehicle- and memantine-treated WT transcriptomes (WT-M/V transcripts) to determine whether early, chronic suppression of NMDARs in WT mice induces any transcriptomic changes, despite its lack of effect on synaptic (NMDAR) and behavioral phenotypes in these mice (Chung et al., 2019). WT-M/V transcripts were positively enriched for synapse-related gene sets, which clustered into synapse-related functions (Figures 5A,B, Supplementary Figure 6, and Supplementary Table 6). WT-M/V transcripts were also negatively enriched for ECM-related gene sets, with ECM-related clustering (Figures 5A,B). These results suggest that early memantine treatment upregulates synaptic genes and downregulates ECM genes in WT mice, a pattern that bears similarity to that observed for Veh-K/W transcripts (upregulated synaptic genes and downregulated ribosome, mitochondria, and ECM genes), although changes in ribosomal and mitochondrial genes were not prominent.

We next compared WT-M/KO-V transcripts to determine whether early, chronic memantine treatment of WT mice induces transcriptomic changes similar to those induced by genetic *Shank2* deletion. Intriguingly, ribosome/mitochondria-related genes showed strong positive enrichment, but negative enrichments among these transcripts were insignificant (Figures 5C,D, Supplementary Figure 7, and Supplementary Table 6). These results suggest that memantine treatment in WT mice compared with *Shank2* gene deletion leads to both similar (synaptic upregulation and ECM downregulation) and distinct (ribosome/mitochondria downregulation only by *Shank2* gene deletion) transcriptomic changes.

### Genes Contributing to Specific Functional Enrichments

What might be the genes that contributed strongly to the abovementioned enrichments to specific biological functions? Although naming a few specific genes cannot cover the whole biological functions associated with the enriched gene sets and their clusters, we summarized some examples (enrichment details for top gene sets) in Supplementary Table 7 for a better grasp on individual gene functions.

One of the top gene sets positively enriched in Veh-K/W transcripts (postsynaptic membrane; C5-CC domain) included *Htr2a* (5-hydroxytryptamine receptor 2A), *Abi1* (abelson interactor 1), *Efnb2* (ephrin B2), *Grik4* (glutamate ionotropic receptor kainate type subunit 4), and *Slc6a3* [solute carrier family 6 member 3 (a dopamine transporter)]. Most of the genes that significantly contributed to the enrichment for “postsynaptic membrane,” including the top genes described above, were associated with excitatory, inhibitory, or both excitatory and inhibitory synaptic functions (Supplementary Table 7), based on SynGO, an evidence-based expert-curated synapse gene database (Koopmans et al., 2019). A top gene set negatively enriched in Veh-K/W transcripts (mitochondrial protein complex; C5-CC) included *Tomm40l* (translocase of outer mitochondrial membrane 40 like), *Mrps21* (mitochondrial ribosomal protein S21), *Mt-cyb* (mitochondrially encoded cytochrome b), *Mt-nd1* (mitochondrially encoded NADH dehydrogenase 1), and *Mt-co1* (mitochondrially encoded cytochrome c oxidase I). Another negatively enriched gene set (ribosome; C5-CC) included *Mrps21* (mitochondrial ribosomal protein S21), *Mrpl12* (mitochondrial ribosomal protein L12), *Rps17* (ribosomal protein S17), *Mrps36* (mitochondrial ribosomal protein S36), and *Rpl12* (ribosomal protein L12).

In addition, one of the top gene sets positively enriched in KO-M/V transcripts in C5-CC domain (chromatin; C5-CC) included *Muc1* (mucin 1), *Ldb1* (LIM domain binding 1), *Cbx7* (chromobox 7), *Plk2* (polo like kinase 2), and *Actr8* (actin related protein 8). Another positively enriched gene set (U2 type spliceosomal complex; C5-CC) included Sf3b1 (splicing factor 3b subunit 1), *Dhx15* (DEAH-box helicase 15), *Snrpa1* (small nuclear ribonucleoprotein polypeptide A′), *Snrpb* (small nuclear ribonucleoprotein polypeptides B and B1), and *Cwc27* (CWC27 spliceosome associated cyclophilin). A top gene set negatively enriched in KO-M/V transcripts (ECM; C5-CC) included *Col9a3* (collagen type IX alpha 3 chain), *Cd151* (CD151 molecule), *Elfn1* (leucine rich repeat and fibronectin type III, extracellular 1), *Pcsk6* (proprotein convertase subtilisin/kexin type 6), and *Tnc* (tenascin C). These results suggest that the enriched biological functions largely agree with individual gene functions.

RT-qPCR analyses indicated some of these genes show expected up- or downregulations in WT-Veh, KO-Veh, and KO-Mem transcripts. For instance, *Htr2a* that contributed to the positive enrichment of Veh-K/W transcripts for the “postsynaptic membrane” gene set showed an increasing tendency of mRNA levels in KO-Veh transcripts relative to WT-Veh transcripts (Supplementary Figure 8). In addition, *Muc1* and *Sf3b1*, which contributed to the positive enrichment of the KO-M/V transcripts for the “chromatin” and “U2 type spliceosomal complex” gene sets, tended to be upregulated in KO-Mem transcripts relative to KO-Veh transcripts.

### GSEA for ASD-Related/Risk Gene Sets

Transcriptomic alterations associated with ASD have been reported (Garbett et al., 2008; Voineagu et al., 2011; Gupta et al., 2014; Parikshak et al., 2016; Velmeshev et al., 2019), together with gene sets that are distinctly up- or downregulated in ASD (ASD-related gene sets), including DEG_Up_Voineagu, Co-Exp_Up_M16_Voineagu, DEG_Down_Voineagu, and Co-Exp_Down_M12_Voineagu (cortical samples with the age range of 2–56) (Voineagu et al., 2011; Werling et al., 2016) (list of genes in these gene sets are summarized in Supplementary Table 8). Previous studies have also established ASD-risk gene sets, including SFARI genes (all) (Abrahams et al., 2013), SFARI (high confidence, belonging to category 1) (Abrahams et al., 2013),^5^ FMRP targets (Darnell et al., 2011; Werling et al., 2016), De Novo Missense (protein-disrupting or missense rare *de novo* variants) (Iossifov et al., 2014; Werling et al., 2016), De Novo Variants (protein-disrupting rare *de novo* variants) (Iossifov et al., 2014; Werling et al., 2016), and AutismKB (Autism KnowledgeBase) (Xu et al., 2012; Yang et al., 2018; Supplementary Table 8). The genes in these ASD-risk gene sets tend to be downregulated in ASD, likely through missense, non-sense, splice-site, frame-shift, and deletion mutations. We thus tested whether early, chronic memantine treatment induced ASD-related transcriptomic changes in WT and *Shank2*-KO mice, by performing GSEA for these ASD-related/risk gene sets and, as inputs, the entire lists of genes with transcriptional changes ranked by *p*-values from Veh-K/W, Mem-K/W, KO-M/V, and WT-M/V transcript groups.

**FIGURE 6:**
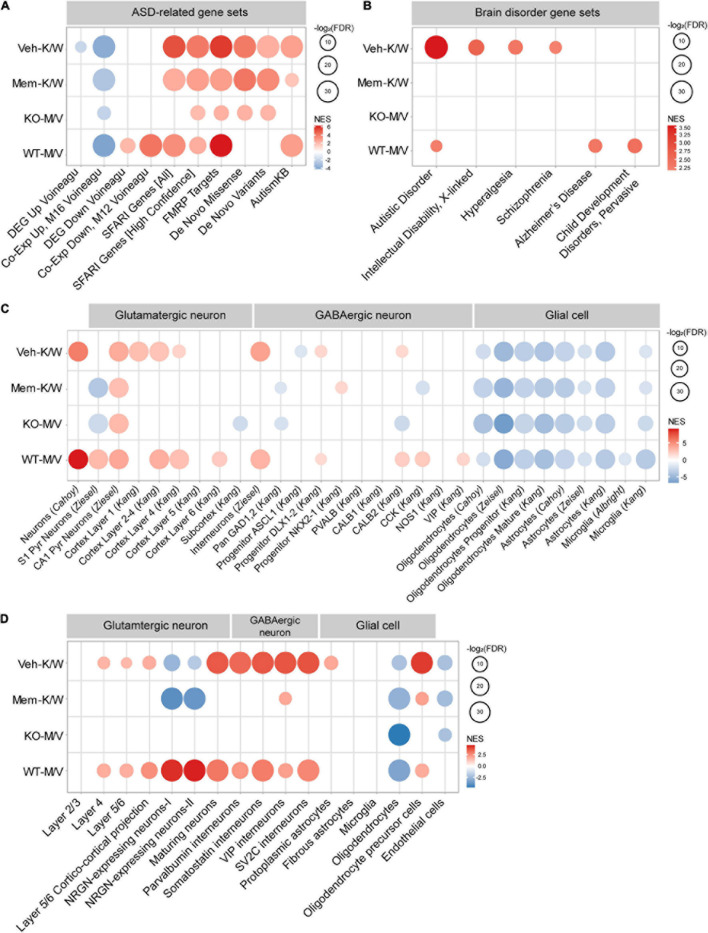
Gene Set Enrichment Analysis of Veh-K/W, Mem-K/W, KO-M/V, and WT-M/V transcripts for ASD- and cell-type–related gene sets. **(A,B)** GSEA results for four transcript groups (Veh-K/W, Mem-K/W, KO-M/V, and WT-M/V) showing enrichment patterns for ASD-related/risk and brain disorder-related (DisGeNet) gene sets, including ASD-related gene sets that are upregulated in ASD (DEG_Up_Voineagu and Co-Exp_Up_M16_Voineagu) and downregulated in ASD (DEG_Down_Voineagu and Co-Exp_Down_M12_Voineagu) and ASD-risk gene sets that are likely to be downregulated in ASD [SFARI (all) SFARI (high confidence), FMRP targets, De Novo Missense, De Novo Variants, and AutismKB]. (*n* = 3 mice for WT-Veh, WT-Mem, KO-Veh, and KO-Mem, FDR < 0.05). **(C)** GSEA results for four transcript groups (Veh-K/W, Mem-K/W, KO-M/V, and WT-M/V) showing enrichment patterns for specific cell-type-specific gene sets. (*n* = 3 mice for WT-Veh, WT-Mem, KO-Veh, and KO-Mem, FDR < 0.05). **(D)** GSEA results for four transcript groups (Veh-K/W, Mem-K/W, KO-M/V, and WT-M/V) showing enrichment patterns for single-cell RNA-Seq-based cell-type-specific gene sets. [*n* = 3 mice (WT-Veh, WT-Mem, KO-Veh, KO-Mem), FDR < 0.05].

Veh-K/W transcripts (baseline WT–KO difference) were negatively enriched for gene sets that are upregulated in ASD (DEG_Up_Voineagu and Co-Exp_Up_M16_Voineagu) but were not enriched for gene sets that are downregulated in ASD (DEG_Down_Voineagu and Co-Exp_Down_M12_Voineagu) (Figure 6A). This pattern is opposite in many respects to the transcriptomic changes that occur in ASD, and is thus here referred to as “reverse-ASD.” In addition, Veh-K/W transcripts were positively enriched for all six tested ASD-risk gene sets [SFARI genes (all) SFARI genes (high confidence), FMRP targets, De Novo Missense, De Novo Variants, and AutismKB; Figure 6A] – again, a reverse-ASD pattern. These results suggest that *Shank2* KO in mice leads to reverse-ASD transcriptomic changes.

KO-M/V transcripts (memantine effects within *Shank2*-KO mice) displayed a relatively weak reverse-ASD pattern, based on the number of enriched gene sets and the extent of enrichment (NES and FDR values), compared with those among Veh-K/W transcripts. Co-Exp-Up was negatively enriched (but DEG_Up was not), and NES/FDR values for ASD-risk gene sets [SFARI genes (all), FMRP targets, and AutismKB] were decreased (Figure 6A). In addition, Mem-K/W transcripts displayed greater weakening in the reverse-ASD pattern (Figure 6A). These results suggest that memantine treatment weakens reverse-ASD transcriptomic patterns in *Shank2*-KO mice.

Among WT-M/V transcripts (memantine effects on WT mice), transcriptomic changes were reverse-ASD, with negative enrichment for Co-Exp_Up, positive enrichment for both DEG_Down and Co-Exp_Down, and positive enrichment for four ASD-risk gene sets [SFARI genes (all), SFARI (high confidence), FMRP targets, and AutismKB] (Figure 6A). These results collectively suggest that (1) *Shank2*-KO mice show a baseline reverse-ASD transcriptomic pattern compared with WT mice, (2) memantine-treated *Shank2*-KO mice show a weakened reverse-ASD transcriptomic pattern compared with vehicle-treated *Shank2*-KO mice, and (3) early memantine treatment of WT mice induces reverse-ASD transcriptomic changes.

Gene Set Enrichment Analysis performed for ASD versus other neuropsychiatric disorders using DisGeNet gene sets^6^ (Pinero et al., 2020) revealed that Veh-K/W transcripts were positively enriched for ASD and moderately enriched for X-linked intellectual disability, hyperalgesia, and schizophrenia (Figure 6B). In contrast, KO-M/V transcripts were not enriched for any of these diseases (ASD, X-linked), and a similar lack of disease association was observed for Mem-K/W transcripts. These results are in line with the abovementioned weakening of reverse-ASD transcriptomic patterns in memantine-treated *Shank2*-KO mice, based on enrichment for ASD-related/risk gene sets. Notably, WT-M/V transcripts were positively, albeit moderately, enriched for gene sets associated with ASD, Alzheimer’s disease and child pervasive developmental disorders, again in line with the positive enrichment of ASD-related/risk gene sets among WT-M/V transcripts.

### GSEA of WT-*Shank2*-KO Transcripts Relative to ASD-Related Cell-Type–Specific Gene Sets

Autism spectrum disorder has been associated with cell-type-specific transcriptomic changes, including downregulation of neuron- and oligodendrocyte-related genes and upregulation of astrocyte- and microglia-related genes (Voineagu et al., 2011; Werling et al., 2016). We thus tested if Veh-K/W, Mem-K/W, KO-M/V, and WT-M/V transcript groups are enriched for cell-type-specific gene sets (Albright and Gonzalez-Scarano, 2004; Cahoy et al., 2008; Kang et al., 2011; Zeisel et al., 2015; Werling et al., 2016; Velmeshev et al., 2019, 2020) (gene set details in Supplementary Table 8).

Veh-K/W transcripts (baseline WT–KO difference) were positively enriched for neuron (glutamatergic and GABAergic)-related gene sets, with stronger enrichment in superficial layers (layers 1–4; glutamatergic neurons) relative to deep layers (layers 5 and 6), and negatively enriched for astrocyte/microglia-related gene sets (Figure 6C). Although these Veh-K/W transcripts were also negatively enriched for oligodendrocyte-related gene sets (ASD-like), the overall enrichment pattern was largely opposite to that observed in ASD (reverse-ASD), in line with the reverse-ASD enrichment pattern of Veh-K/W transcripts for ASD-related/risk gene sets.

KO-M/V transcripts (memantine effects on KO mice) showed substantially reduced enrichment for neuron-related gene sets, in particular for superficial cortical glutamatergic neurons, in contrast to the upregulated expression of neuronal genes among Veh-K/W transcripts, although negative enrichment for oligodendrocyte-, astrocyte-, and microglia-related gene sets remained unaltered (Figure 6C). Mem-K/W transcripts also showed similarly weakened enrichment for neuron-related gene sets. WT-M/V transcripts showed largely positive enrichment for neuron-related gene sets, with strong enrichment for glutamatergic neurons in superficial cortical layers.

When the four transcript groups (Veh-K/W, Mem-K/W, KO-M/V, and WT-M/V) were tested for cell-type-specific genes validated by single-cell RNA-Seq results of human cortical tissues (Velmeshev et al., 2019), Veh-K/W transcripts were positively enriched in neurons, more strongly in GABA neurons relative to glutamate neurons, and in oligodendrocyte precursors (Figure 6D), indicative of a reverse-ASD pattern. This reverse-ASD patterns was largely eliminated in Mem-K/W and KO-M/V transcripts, partly similar to the results for ASD-related/risk gene sets. In addition, WT-M/V transcripts showed largely reverse-ASD patterns. However, the stronger enrichment of Veh-K/W transcripts for superficial cortical glutamate neurons were not observed. Notably, gene sets associated with neurogranins, a regulator of synaptic function and marker of neurodegeneration (Diez-Guerra, 2010; Lista and Hampel, 2017), were distinctly enriched in Veh-K/W and WT-M/V transcripts.

These results collectively suggest that (1) *Shank2*-KO mice show reverse-ASD baseline transcriptomic changes in both neurons and glial cells (astrocytes/microglia), (2) memantine treatment in *Shank2*-KO mice weakens reverse-ASD transcriptomic patterns in neurons but not in glial cells.

## Discussion

In the present study, we compared four sets of transcriptomes in WT and *Shank2*-KO mice treated early and chronically with vehicle or memantine to glean mechanistic insights into how early memantine treatment rescues phenotypic deficits in *Shank2*-KO mice. We found substantial changes in transcriptomic patterns involving synapse-, ribosome-, mitochondria-, ECM-, chromatin-, and ASD-related genes (summarized in Supplementary Figure 9). In addition, we found that early, chronic memantine treatment of WT mice, in which memantine does not induce detectable synaptic or behavioral changes (Chung et al., 2019), induces transcriptomic changes involving synaptic and ECM functions.

Whether functional enrichment of a particular gene set or a group of gene sets is indicative of a key pathophysiological mechanism in *Shank2*-KO mice or merely reflects compensatory responses remains unclear. However, additional enrichment patterns for ASD-related gene sets (ASD-related/risk and cell-type–specific), revealing reverse-, and ASD-like tendencies of the transcripts, provide hints on whether the identified biological functions represent pathological mediators or compensatory responses. In addition, memantine-induced changes in enrichment patterns for biological functions and ASD tendency, which were often inversely correlated with those observed in vehicle-treated *Shank2*-KO mice, provide further insights into the roles of up-/downregulated genes. We highlight these aspects in the following discussion, although it should be pointed out that these aspects were not validated by functional experiments, and thus care should be taken in interpreting the correlative results.

The first transcriptomic change to note is the baseline difference between vehicle-treated WT and *Shank2*-KO mice (Veh-K/W transcripts), which showed positive enrichment for synapse-related gene sets. Given that Shank2 is a key excitatory postsynaptic scaffolding protein (Kim and Sheng, 2004; Grabrucker et al., 2011; Mossa et al., 2017) and that *Shank2*-KO mice show decreased NMDAR function at ∼P25 (Won et al., 2012; Chung et al., 2019), the upregulation of synaptic genes likely represents compensatory changes that serve to normalize the decreased NMDAR function at excitatory synapses. In support of this possibility, Veh-K/W transcripts showed a reverse-ASD tendency, whereas Mem-K/W transcripts showed weakened synaptic upregulation and reverse-ASD tendency. In addition, WT-M/V transcripts showed upregulation of synaptic genes and a reverse-ASD tendency.

Veh-K/W transcripts also displayed negative enrichment for ribosome-related gene sets. Given the known reciprocal relationship between synaptic proteins and protein synthesis in ASD (Santini and Klann, 2014), it is possible that decreased NMDAR function may suppress mTOR signaling and translation of related proteins known to underlie various neurodevelopmental and psychiatric disorders, including ASD (Hoeffer and Klann, 2010; Costa-Mattioli and Monteggia, 2013; Borrie et al., 2017; Switon et al., 2017; Winden et al., 2018). In line with this possibility, a deficiency or duplication of Shank3 (a Shank2 relative) has been linked to altered mTOR signaling in rodent and human neurons (Bidinosti et al., 2016; Lee et al., 2017). More directly, an mTOR signaling-related gene set was significantly and positively enriched in Veh-K/W transcripts, but this enrichment was insignificant in both KO-M/V and Mem-K/W transcripts (Supplementary Figure 10). In addition, the downregulation of ribosome-related genes in Veh-K/W transcripts was no longer observed in KO-M/V transcripts (with a weakened reverse-ASD tendency).

Veh-K/W transcripts also displayed downregulation of mitochondria- and ECM-related genes. Mitochondrial dysfunction has been associated with synapse dysregulation (Li et al., 2004; Vos et al., 2010; Sheng and Cai, 2012; Lee et al., 2018) as well as ASD (Hollis et al., 2017; Frye, 2020; Rojas-Charry et al., 2021). It is possible that the upregulation of synaptic genes in the context of a reverse-ASD pattern in *Shank2*-KO mice might be related to decreased mitochondrial gene expression. However, mitochondria downregulation continued to be observed in KO-M/V and Mem-K/W transcripts (weakened reverse-ASD tendency) in addition to Veh-K/W transcripts (strong reverse-ASD tendency), despite that NMDAR function is rescued by memantine treatment in *Shank2*-KO mice (Grabrucker et al., 2021), suggesting that downregulation of mitochondria-related genes may be reflective of some non-NMDAR functions and non-social behavioral deficits (i.e., repetitive behaviors) that are not rescued by memantine treatment (Chung et al., 2019). Another notable transcriptomic change in Veh-K/W transcripts was the parallel downregulation of mitochondrial and ribosomal genes. This might be attributable to the fact that mitochondrial function must be adjusted to match the energy consumption involved in protein translation (Morita et al., 2015).

Products of ECM-related genes are known to regulate synapse/neuronal development and function (Frischknecht et al., 2014; Condomitti and de Wit, 2018; Song and Dityatev, 2018; Fawcett et al., 2019) and have been implicated in various cognitive and neuropsychiatric disorders, including ASD, Alzheimer’s disease, schizophrenia, and fragile X syndrome (Condomitti and de Wit, 2018; Fawcett et al., 2019). The expression of ECM-related genes was decreased in Veh-K/W, KO-M/V, and WT-M/V transcript groups, a decrease that appeared to be inversely correlated with an increase in synaptic gene expression in two transcript groups (Veh-K/W and WT-M/V but not KO-M/V), both of which show strong reverse-ASD transcriptomic tendency. It is possible that the decrease in ECM expression may help increase synaptic function in *Shank2*-KO mice and memantine-treated WT mice. In line with this idea, a recent study showed that targeted degradation of the ECM with chondroitinase ABC induces an increase in the number of excitatory synapses (Dembitskaya et al., 2021).

The chromatin upregulation in KO-M/V and Mem-K/W transcripts (weakened reverse-ASD tendency) but not in Veh-K/W transcripts (strong reverse-ASD tendency) suggests a role for this upregulation in mediating memantine-dependent phenotypic rescue. Previous studies have associated chromatin remodelers such as CHD8 and ARID1B with ASD-risk and validated them in animal models (De Rubeis et al., 2014; Barnard et al., 2015; Moffat et al., 2021). In addition, alterations in chromatin functions known to regulate neural development and cognitive brain function (Ronan et al., 2013; Goodman and Bonni, 2019) have been observed in mouse models of ASD and human neurons carrying ASD-risk mutations, including *Shank3*-mutant mice (Zhu et al., 2014; Ma et al., 2018; Qin et al., 2018; Wang et al., 2019; Zhang et al., 2021) and human *SHANK2*-KO neurons (Zaslavsky et al., 2019). Given the broad impacts of chromatin remodelers in regulating the expression of various genes, chromatin upregulation in memantine-treated *Shank2*-KO mice might be involved in the regulation of various targets genes (i.e., mitochondria/ECM-related) that rescue synaptic and behavioral phenotypes.

A GSEA of transcripts for ASD-related/risk gene sets revealed a strong reverse-ASD tendency for Veh-K/W transcripts. This reverse-ASD tendency was weakened in both KO-M/V and, to an even greater extent, in Mem-K/W transcripts, where the strong positive enrichment for SFARI genes observed in Veh-K/W transcripts was no longer significant. Therefore, it is tempting to speculate that changes in the expression of SFARI genes are more important relative to other ASD-risk genes such as FMRP targets for memantine-dependent phenotypic rescue in *Shank2*-KO mice, and that these SFARI genes likely involve the gene-set functions that are strongly distinct between Veh-K/W and Mem-K/W transcripts, such as synapse/ECM and chromatin/actin-related functions.

With regard to cell-type–related gene sets, Veh-K/W transcripts showed neuronal upregulation and astrocytic/microglial downregulation – a strongly reverse-ASD pattern. In contrast, KO-M/V and Mem-K/W transcripts showed a weakened reverse-ASD tendency, as supported by moderate to strong decreases in neuronal upregulation, depending on gene cell-type-specific gene sets used. These memantine-dependent changes (Figures 6C,D) are stronger than those observed in GSEA results using ASD-related/risk genes (Figure 6A), suggesting that cell-type-specific transcriptomic changes might play more important roles, relative to changes in ASD-related/risk genes. Astrocytic/microglial downregulations, however, remained unchanged compared with Veh-K/W transcripts. These results collectively suggest a decreased need for compensatory neuronal upregulation in memantine-treated *Shank2*-KO mice and suggest a key role for neuronal (but not glial) gene expression in the phenotypic rescue.

Notably, both Veh-K/W and KO-M/V (or Mem-K/W) transcripts were more strongly enriched for glutamatergic neurons in superficial cortical layers relative to those in deep cortical layers, although this was true in a subset of cell-type-specific gene sets. This is in agreement with previous results showing that multiple ASD-risk genes and gene modules are enriched in superficial cortical layers (layers 2–4) and that key ASD-risk genes, including *SHANK2*, are strongly expressed in superficial cortical layers (Parikshak et al., 2013; Velmeshev et al., 2019). Whether superficial-layer glutamatergic neurons in *Shank2*-KO mice preferentially undergo the abovementioned changes in the expression of ASD-related/risk genes and chromatin/mitochondria/ECM/actin-related genes remains to be determined.

Lastly, WT-M/V transcripts were positively enriched for synapse-related gene sets and negatively enriched for ECM-related gene sets. These changes seem to represent reverse-ASD transcriptomic changes, based on enrichment patterns for ASD-related/risk and cell-type-specific gene sets. Veh-K/W transcripts also showed similar synaptic upregulation, ECM downregulation, and a strong reverse-ASD tendency. However, Veh-K/W transcripts are unique in that ribosomal/mitochondrial genes were also downregulated, suggesting that pharmacological inhibition of NMDARs and genetic deletion of *Shank2* induce different transcriptomic changes. In line with this, WT-M/V and Veh-K/W transcripts displayed both shared and distinct patterns of enrichment of ASD-related/risk and cell-type-specific gene sets, despite the fact that transcriptomic changes in both cases are reverse-ASD.

In summary, our results suggest that early, chronic memantine treatment induces transcriptomic changes involving chromatin, mitochondria, ECM, and actin functions in *Shank2*-KO mice as well as weakening of the reverse-ASD tendency involving ASD-related/risk and cell-type-specific genes.

## Data Availability Statement

Raw RNA-Seq results are available as GSE171931 at Gene Expression Ombinus (GEO) and National Center for Biotechnology Information (NCBI).

## Ethics Statement

The animal study was reviewed and approved by the Committee of Animal Research at KAIST.

## Author Contributions

Y-EY, WK, HKi, CC, SH, SL, and HKa performed RNA-Seq analyses. JP and YC performed statistical power analysis. HKa and EK wrote the manuscript. All authors contributed to the article and approved the submitted version.

## Conflict of Interest

The authors declare that the research was conducted in the absence of any commercial or financial relationships that could be construed as a potential conflict of interest.

## Publisher’s Note

All claims expressed in this article are solely those of the authors and do not necessarily represent those of their affiliated organizations, or those of the publisher, the editors and the reviewers. Any product that may be evaluated in this article, or claim that may be made by its manufacturer, is not guaranteed or endorsed by the publisher.

## Footnotes

1
http://www.informatics.jax.org/homology.shtml

2
http://software.broadinstitute.org/gsea

3
http://software.broadinstitute.org/gsea/msigdb

4
https://www.gsea-msigdb.org/gsea/index.jsp

5
https://gene.sfari.org/

6
https://www.disgenet.org/

## Supplementary Material

Supplementary Figure 1 Results of PCA analysis for the four RNA sample groups and statistical power analysis for sample size. **(A)** Principal component analysis (PCA) plots for WT-Veh, WT-Mem, KO-Veh, and KO-Mem transcriptomes. **(B)** Statistical power calculated for sample size of 3, 6, and 9 per group in each of the six transcript comparisons (Veh-K/W, Mem-K/W, KO-M/V, K-M/W-V, WT-M/V, and WT-M/KO-V).

Supplementary Figure 2 Gene Set Enrichment Analysis results for Veh-K/W transcripts in C5-BP (biological process) and C5-MF (molecular function) domains. **(A,B)** GSEA results for Veh-K/W transcripts showing a list of the top five positively (red) and negatively (blue) enriched gene sets in the C5-BP domain **(A)** and their integrated visualization generated using the Cytoscape EnrichmentMap App **(B)**. [*n* = 3 mice (WT-Veh, KO-Veh), FDR < 0.05]. **(C,D)** GSEA results (**C**; top five lists) and their integrated visualization using EnrichmentMap **(D)** for Veh-K/W transcripts using gene sets in the C5-MF domain. [*n* = 3 mice (WT-Veh, KO-Veh), FDR < 0.05].

Supplementary Figure 3 Gene Set Enrichment Analysis results for Mem-K/W transcripts in C5-BP and C5-MF domains. **(A,B)** GSEA results (**A**; top five lists) and their integrated visualization (**B**; Cytoscape EnrichmentMap) for Mem-K/W transcripts using gene sets in the C5-BP domain. [*n* = 3 mice (WT-Mem, KO-Mem), FDR < 0.05]. **(C,D)** GSEA results (**C**; top five lists) and their integrated visualization (**D**; Cytoscape EnrichmentMap) for Mem-K/W transcripts using gene sets in the C5-MF domain. [*n* = 3 mice (WT-Mem, KO-Mem), FDR < 0.05].

Supplementary Figure 4 Gene Set Enrichment Analysis results for KO-M/V transcripts in C5-BP and C5-MF domains. **(A,B)** GSEA results (**A**; top five lists) and their integrated visualization (**B**; Cytoscape EnrichmentMap) for KO-M/V transcripts using gene sets in the C5-BP domain. [*n* = 3 mice (KO-Veh, KO-Mem), FDR < 0.05]. **(C,D)** GSEA results (**C**; top five lists) and their integrated visualization (**D**; Cytoscape EnrichmentMap) for KO-M/V transcripts using gene sets in the C5-MF domain. [*n* = 3 mice for (KO-Veh, KO-Mem), FDR < 0.05].

Supplementary Figure 5 Gene Set Enrichment Analysis results for KO-M/WT-V transcripts in C5-BP and C5-MF domains. **(A,B)** GSEA results (**A**; top five lists) and their integrated visualization (**B**; Cytoscape EnrichmentMap) for KO-M/WT-V transcripts using gene sets in the C5-BP domain. [*n* = 3 mice (WT-Veh, KO-Mem), FDR < 0.05]. **(C,D)** GSEA results (**C**; top five lists) and their integrated visualization (**D**; Cytoscape EnrichmentMap) for KO-M/WT-V transcripts using gene sets in the C5-MF domain. [*n* = 3 mice (WT-Veh, KO-Mem), FDR < 0.05].

Supplementary Figure 6 Gene Set Enrichment Analysis results for WT-M/V transcripts in C5-BP and C5-MF domains. **(A,B)** GSEA results (**A**; top five lists) and their integrated visualization (**B**; Cytoscape EnrichmentMap) for WT-M/V transcripts using gene sets in the C5-BP domain. [*n* = 3 mice (WT-Veh, WT-Mem), FDR < 0.05]. **(C,D)** GSEA results (**C**; top five lists) and their integrated visualization (**D**; Cytoscape EnrichmentMap) for WT-M/V transcripts using gene sets in the C5-MF domain. [*n* = 3 mice (WT-Veh, WT-Mem), FDR < 0.05].

Supplementary Figure 7 Gene Set Enrichment Analysis results for WT-M/KO-V transcripts in C5-BP and C5-MF domains. **(A,B)** GSEA results (**A**; top five lists) and their integrated visualization (**B**; Cytoscape EnrichmentMap) for WT-M/KO-V transcripts using gene sets in the C5-BP domain. [*n* = 3 mice (WT-M, KO-Veh), FDR < 0.05]. **(C,D)** GSEA results (**C**; top five lists) and their integrated visualization (**D**; Cytoscape EnrichmentMap) for WT-M/KO-V transcripts using gene sets in the C5-MF domain. [*n* = 3 mice (WT-M, KO-Veh), FDR < 0.05].

Supplementary Figure 8 RT-qPCR validation of the key genes contributing to the enrichments of Veh-K/W and KO-M/W transcript groups for gene sets in the C5-CC domain. **(A)** List of the top genes contributing most strongly to the gene-set enrichments shown in Supplementary Table 7 (i.e., postsynaptic density for Veh-K/W transcripts) and their transcriptional changes in the four transcript comparisons (Veh-K/W, Mem-K/W, KO-M/V, and WT-M/V). The light-green shades indicate significant (adjusted *p*-value < 0.05) differential mRNA expression in the indicated transcript groups. **(B)** RT-qPCR results for each gene from WT-Veh, KO-Veh, and KO-Mem samples for comparison of WT-Veh vs. KO-Veh (for top genes from Veh-K/W transcripts), or KO-Veh vs. KO-Mem (for top gene from KO-M/V transcripts).

Supplementary Figure 9 Summary of the GSEA results. Summary of the GSEA results of all four sets of transcripts (Veh-K/W, Mem-K/W, KO-M/V, and WT-M/V) for biological function (CC, BP, and MF)-related, ASD-related (DEG/Co-Exp_Up/Down), ASD-risk [SFARI (all and high-confidence), FMRP target, De Novo Missense, De Novo Variants, and AutismKB], and cell-type-specific gene sets. Positive, insignificant, and negative enrichments for individual, or groups of, gene sets are indicated by red, black, and blue colors in texts/numbers. Enrichment results for single-cell-based cell-type-specific gene sets were summarized here for redundancy.

Supplementary Figure 10 Gene Set Enrichment Analysis results of four transcript groups (Veh-K/W, Mem-K/W, KO-M/V, and WT-M/V) for KEGG signaling pathway gene sets. GSEA results for all four transcript groups (Veh-K/W, Mem-K/W, KO-M/V, and WT-M/V) showing enrichment patterns for selected KEGG biological-pathway gene sets, including mTOR signaling. [*n* = 3 mice (Veh-K/W, Mem-K/W, KO-M/V, WT-M/V), FDR < 0.05].

Supplementary Table 1 Total RNA-Seq results of forebrain transcriptomes from early and chronically vehicle/memantine-treated WT and *Shank2*-KO mice.

Supplementary Table 2 Lists of up- and downregulated DEGs in Veh-K/W, Mem-K/W, KO-M/V, and WT-M/V transcripts.

Supplementary Table 3 Results of Enrichr analysis for DEGs in Veh-K/W and Mem-K/W transcripts (CC, BP, MF, and KEGG). Note that Enrichr analysis for DEGs in WT-M/V and KO-M/V transcript did not yield significant functional annotations in GO terms. Adjusted *p*-values < 0.05.

Supplementary Table 4 Gene Set Enrichment Analysis results (C5-CC, C5-BP, and C5-MF) for Veh-K/W and Mem-K/W transcripts.

Supplementary Table 5 Gene Set Enrichment Analysis results (C5-CC, C5-BP, and C5-MF) for KO-M/V and KO-M/WT-V transcripts.

Supplementary Table 6 Gene Set Enrichment Analysis results (C5-CC, C5-BP, and C5-MF) for WT-M/V and WT-M/KO-V transcripts.

Supplementary Table 7 Details on the top enriched gene sets in Veh-K/W, Mem-KO/WT, and KO-M/V transcript groups, showing information on individual genes contributed to the enrichments such as original rank and running enrichment score. The rank metric score for each gene in GSEA is calculated by using a default signal-to-noise ratio ranking metric. The rank of 14 for HTR2A indicates its rank in the whole-gene input list. A tap also indicates the association of the genes that significantly contributed to the enrichment of Veh-K/W transcripts for “postsynaptic membrane” gene sets with excitatory, inhibitory, and both excitatory and inhibitory synaptic functions.

Supplementary Table 8 List ASD-related/risk and cell-type-specific gene sets used for GSEA.
