# Supplementary material for: Early Chronic Memantine Treatment-Induced Transcriptomic Changes in Wild-Type and Shank2-Mutant Mice
Source: Front Mol Neurosci. 2021 Sep 14;14:712576. doi: 10.3389/fnmol.2021.712576 (PMC8477010; doi:10.3389/fnmol.2021.712576)

Supplementary Figure 1

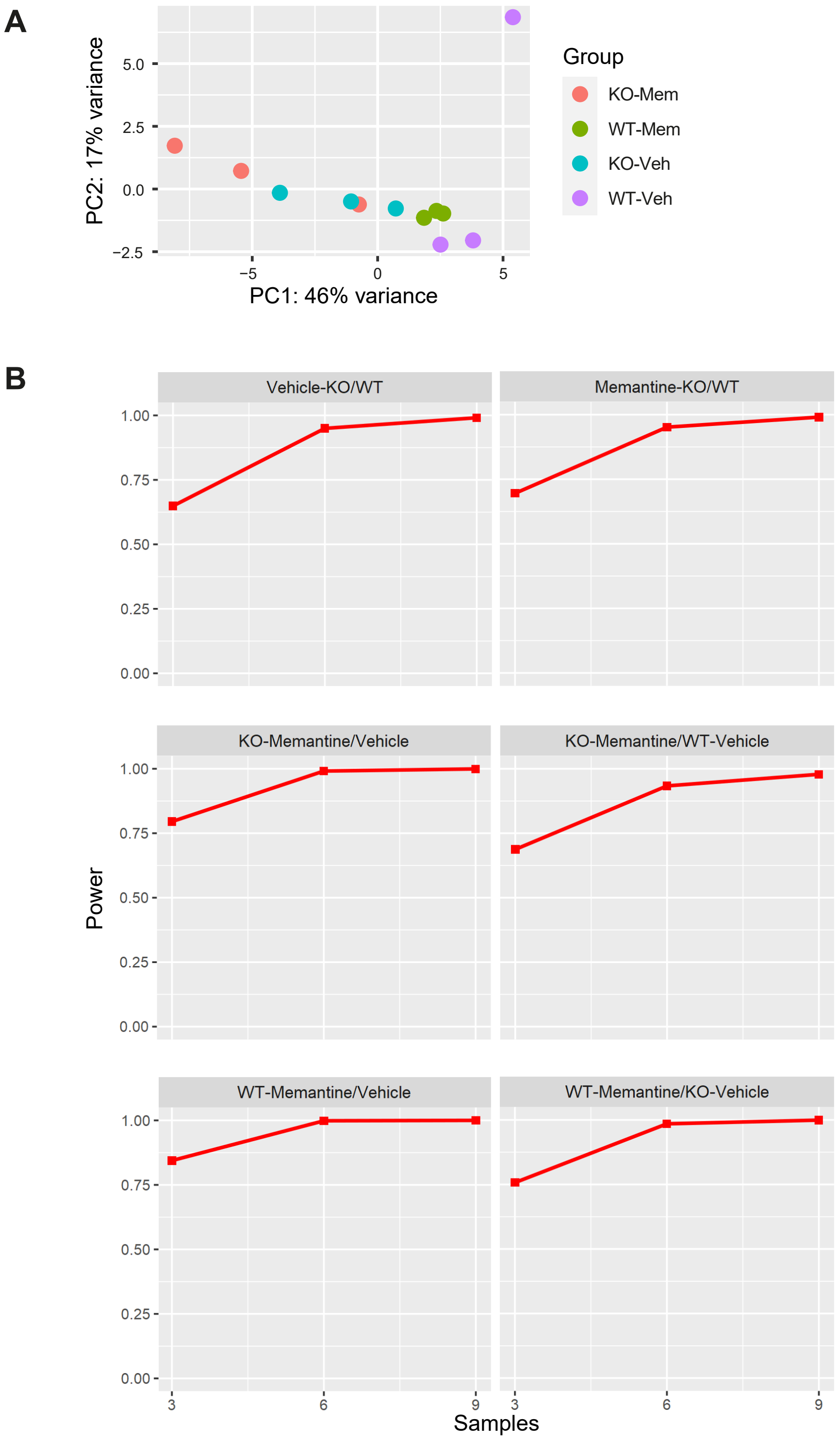

Supplementary Figure 2

A

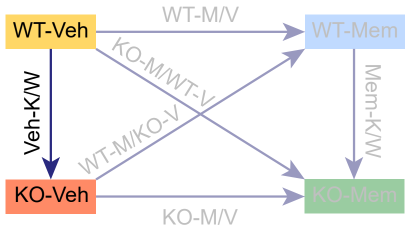

Top5 positively enriched gene sets (Vehicle-K/W, C5-BP)

Name	NES	FDR
Chloride transmembrane transport	2.99	0.003
Rhythmic process	2.98	0.002
Inorganic anion transmembrane transport	2.96	0.001
Regulation of postsynaptic membrane potential	2.95	0.001
Inorganic anion transport	2.87	0.003

Top5 negatively enriched gene sets (Vehicle-K/W, C5-BP)

Name	NES	FDR
Extracellular structure organization	–3.57	0.000
Mitochondrial respiratory chain complex assembly	–3.36	0.000
Mitochondrial translation	–3.27	0.000
Integrated mediated signaling pathway	–3.20	0.002
Electron transport chain	–3.19	0.002

B

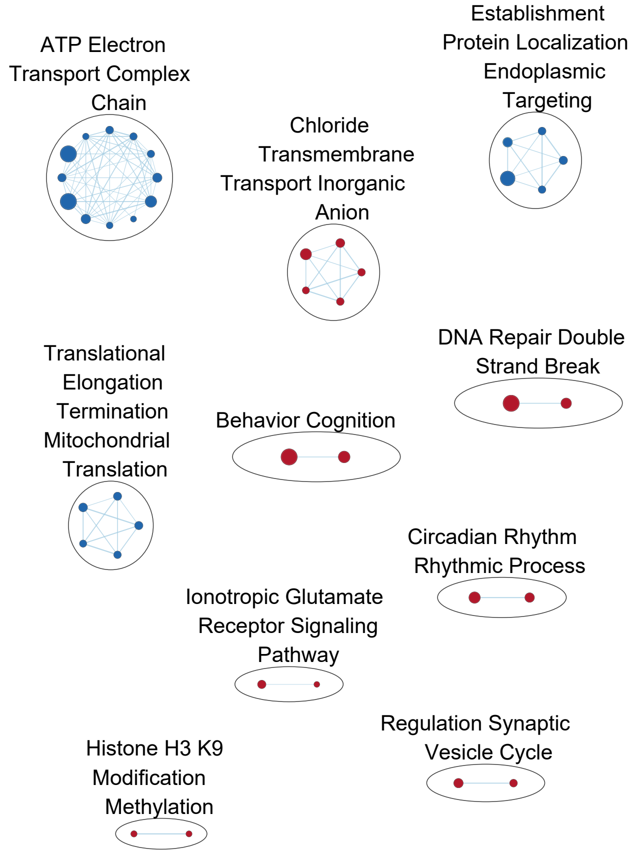

C

Top5 positively enriched gene sets (Vehicle-K/W, C5-MF)

Name	NES	FDR
Transmitter gated channel activity	3.29	0.000
Extracellular ligand ion channel activity	3.28	0.000
Neurotransmitter receptor activity	3.21	0.000
Inorganic anion transmembrane transporter activity	3.00	0.000
Chloride transmembrane transporter activity	2.92	0.001

Top5 negatively enriched gene sets (Vehicle-K/W, C5-MF)

Name	NES	FDR
Structural constituent of ribosome	–3.81	0.000
Extracellular matrix structural constituent	–2.93	0.001
Electron transfer activity	–2.90	0.001
NADH dehydrogenase activity	–2.45	0.016
Oxidoreductase activity acting on NADPH quinone or similar compound as acceptor	–2.38	0.023

D

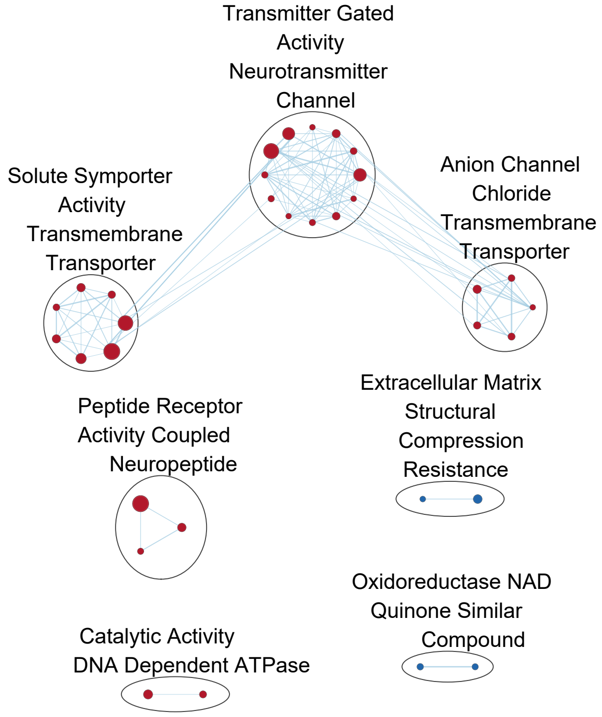

Supplementary Figure 3

A

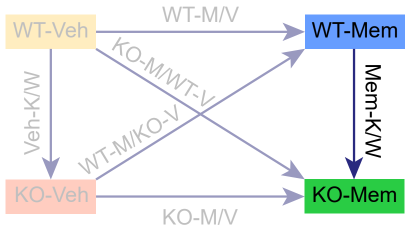

Top5 positively enriched gene sets (Memantine-K/W, C5-BP)

Name	NES	FDR
Covalent chromatin modification	4.18	0.000
Regulation of chromosome organization	3.93	0.000
Regulation of chromatin organization	3.92	0.000
mRNA metabolic process	3.80	0.000
Peptidyl lysine modification	3.76	0.000

Top5 negatively enriched gene sets (Memantine-K/W, C5-BP)

Name	NES	FDR
Oxidative phophorylation	–4.55	0.000
Electron transport chain	–4.11	0.000
Cellular respiration	–3.98	0.000
ATP synthesis coupled electron transport	–3.84	0.000
Establishment of protein localization to endoplasmic reticulum	–3.83	0.000

B

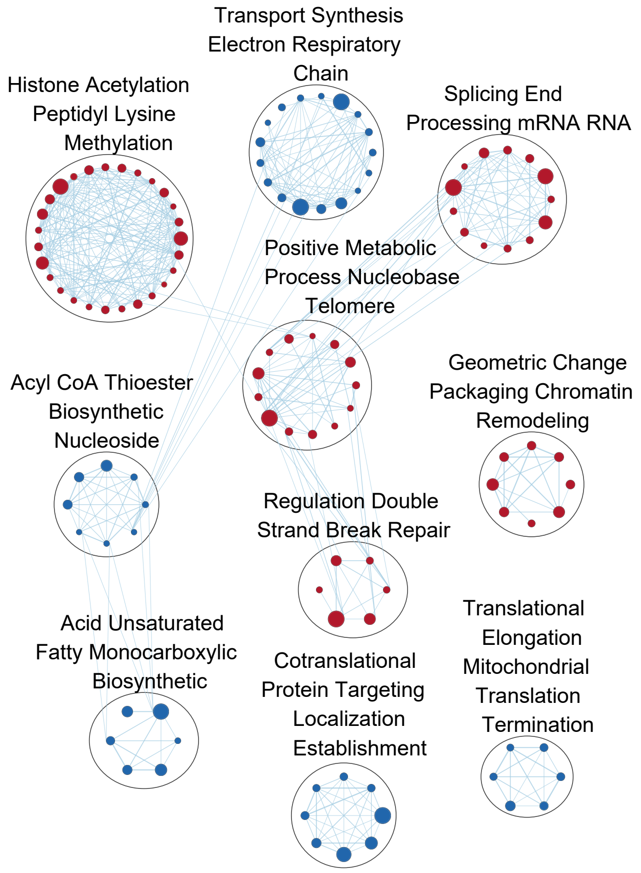

C

Top5 positively enriched gene sets (Memantine-K/W, C5-MF)

Name	NES	FDR
Chromatin binding	4.43	0.000
Proximal promoter sequence specific DNA binding	3.18	0.000
Helicase activity	3.13	0.000
Transcription co-regulator activity	2.98	0.001
Histone binding	2.95	0.001

Top5 negatively enriched gene sets (Memantine-K/W, C5-MF)

Name	NES	FDR
Structural constituent of ribosome	–4.26	0.000
Electron transfer activity	–3.06	0.000
Oxidoreductase activity acting on NADPH quinone or similar compound as acceptor	–2.77	0.002
Oxidoreductase activity acting on NADPH	–2.71	0.002
NADH dehydrogenase activity	–2.66	0.003

D

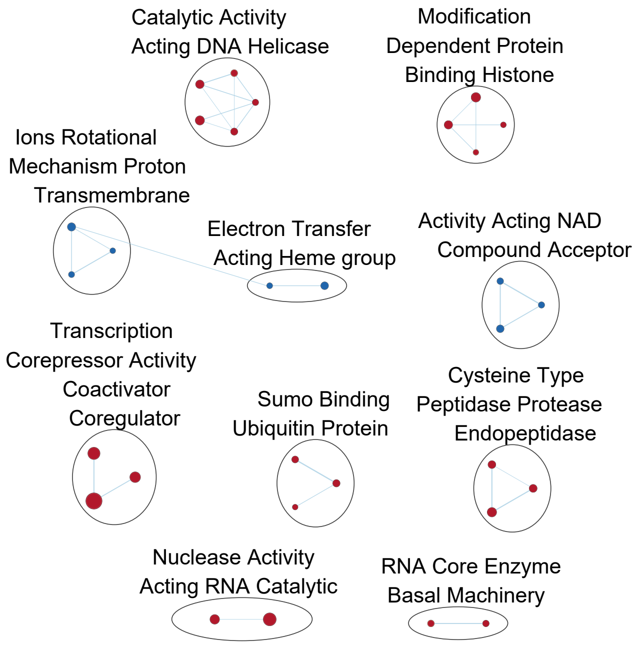

Supplementary Figure 4

A

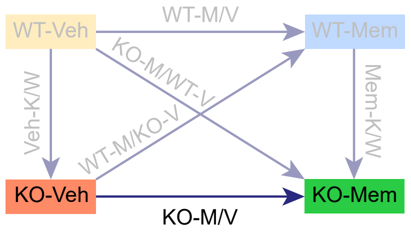

Top5 positively enriched gene sets (KO-M/V, C5-BP)

Name	NES	FDR
mRNA metabolic process	4.69	0.000
Peptidyl lysine modification	4.30	0.000
Covalent chromatin modification	4.10	0.000
RNA splicing	4.10	0.000
RNA splicing via transesterification reactions	4.05	0.000

Top5 negatively enriched gene sets (KO-M/V, C5-BP)

Name	NES	FDR
Adenylate cyclase inhibiting G protein coupled receptor signaling pathway	–3.08	0.000
Monocarboxylic acid metabolic process	–3.03	0.000
Drug metabolic process	–2.85	0.004
Purine containing compound biosynthetic process	–2.78	0.006
Organic hydroxy compound metabolic process	–2.74	0.008

B

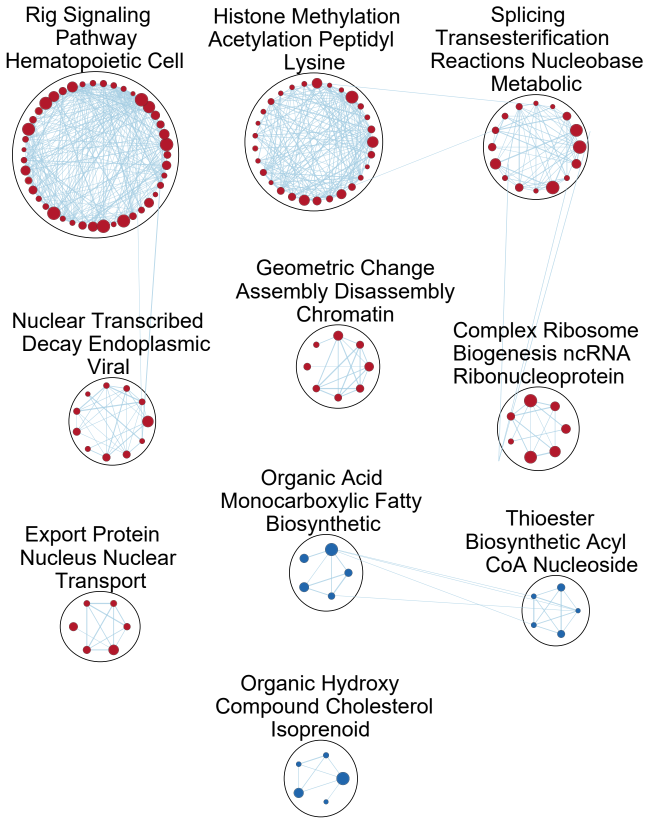

C

Top5 positively enriched gene sets (KO-M/V, C5-MF)

Name	NES	FDR
Chromatin binding	4.82	0.000
Transcription co-regulator activity	3.54	0.000
Transcription co-activator activity	3.50	0.000
Histone binding	3.42	0.000
Proximal promoter sequence specific DNA binding	3.30	0.000

Top5 negatively enriched gene sets (KO-M/V, C5-MF)

Name	NES	FDR
Extracellular matrix structural constituent	–3.12	0.000
Intergrin binding	–2.71	0.002
Hydro lyase activity	–2.61	0.006
Motor activity	–2.53	0.011
Cell adhesion mediator activity	–2.49	0.013

D

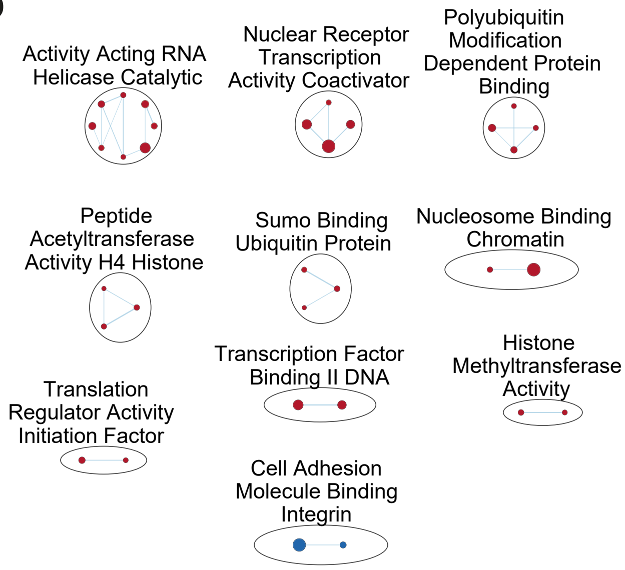

Supplementary Figure 5

A

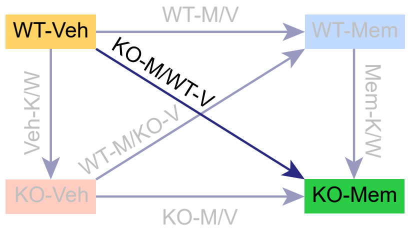

Top5 positively enriched gene sets (KO-M/WT-V, C5-BP)

Name	NES	FDR
Covalent chromatin modification	3.91	0.000
Regulation of chromosome organization	3.78	0.000
Regulation of chromatin organization	3.77	0.000
Regulation of DNA metabolic process	3.61	0.000
Peptidyl lysine modification	3.56	0.000

Top5 negatively enriched gene sets (KO-M/WT-V, C5-BP)

Name	NES	FDR
Extracellular structure organization	–3.74	0.000
Electron transport chain	–3.63	0.000
Oxidatitve phosphorylation	–3.63	0.000
Mitochondrial respiratory chain complex assembly	–3.52	0.000
Resipiratory electron transport chain	–3.19	0.000

B

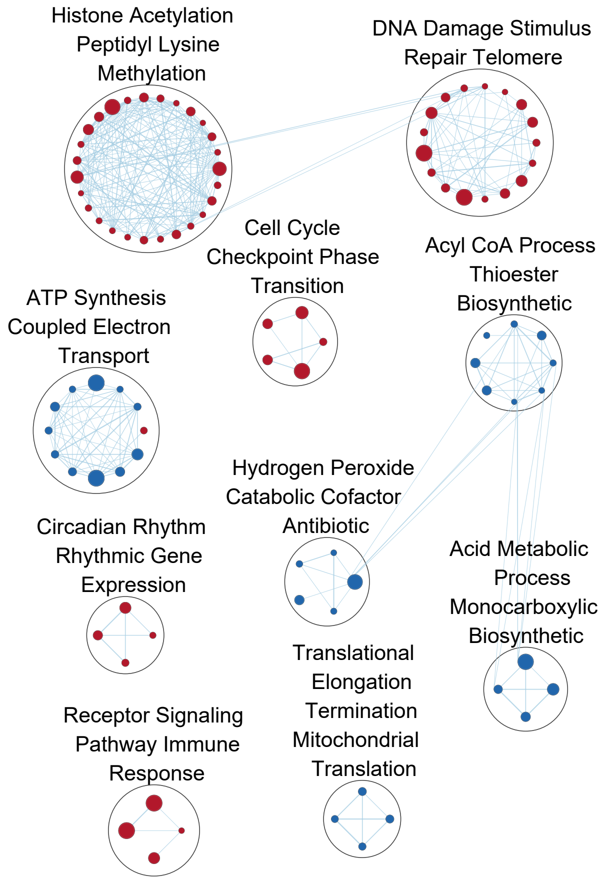

C

Top5 positively enriched gene sets (KO-M/WT-V, C5-MF)

Name	NES	FDR
Chromatin binding	3.65	0.000
Ubiquitin like protein transferase activity	3.51	0.000
Transcription co-regulator activity	3.29	0.000
Transcription co-activator activity	3.07	0.000
Ubiquitin like protein ligase activity	2.98	0.000

Top5 negatively enriched gene sets (KO-M/WT-V, C5-MF)

Name	NES	FDR
Extracellular matrix structural constituent	–3.42	0.000
Structural constituent of ribosome	–3.02	0.000
Integrin binding	–3.01	0.000
Glycosaminoglycan binding	–2.85	0.000
Electron transfer activity	–2.66	0.003

D

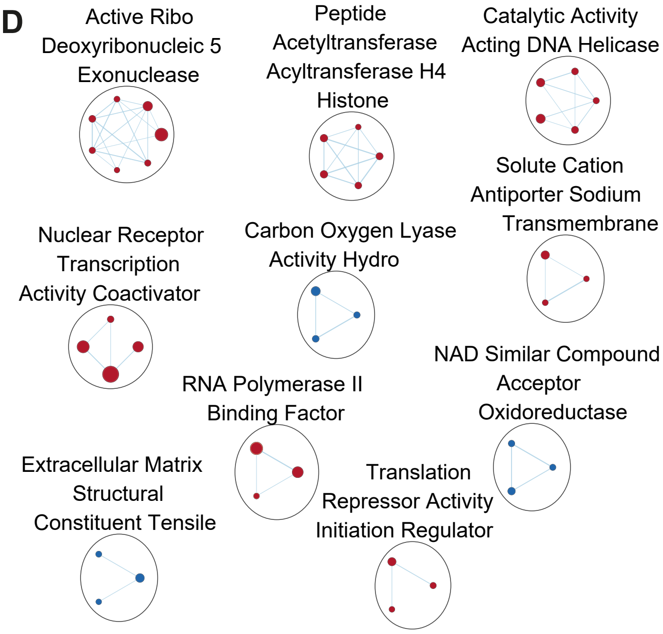

Supplementary Figure 6

A

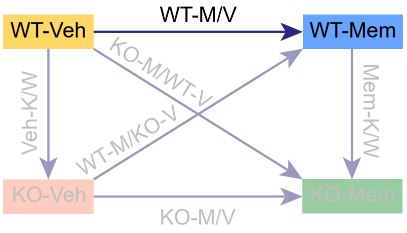

Top5 positively enriched gene sets (WT-M/V, C5-BP)

Name	NES	FDR
Regulation of synapse structure or activity	3.59	0.000
Synaptic vesicle cycle	3.49	0.001
Synapse organization	3.49	0.000
Regulation of trans synaptic signaling	3.31	0.000
Proteosomal protein catabolic process	3.02	0.001

Top5 negatively enriched gene sets (WT-M/V, C5-BP)

Name	NES	FDR
Extracellular structure organization	–3.59	0.000
Collagen fibril organization	–2.88	0.000
Adaptive immune response based on somatic recombination of immune receptors built from immunoglobulin superfamily domains	–2.66	0.000
Adaptive immune response	–2.55	0.000
Positive regulation of interleukin-12 production	–2.53	0.000

B

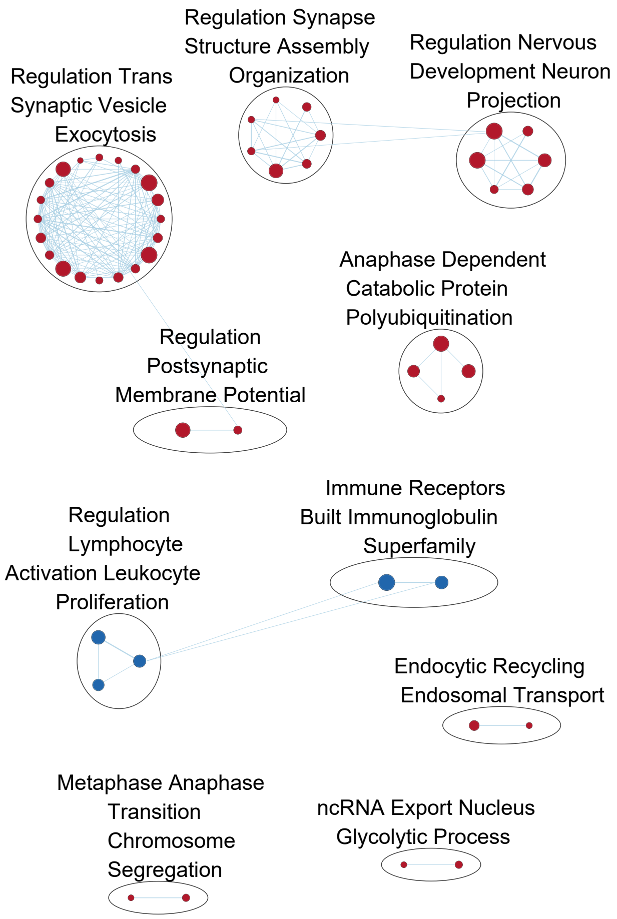

C

Top5 positively enriched gene sets (WT-M/V, C5-MF)

Name	NES	FDR
Ubiquitin like protein transferase activity	2.90	0.000
GTPase activity	2.71	0.006
Monovalent inorganic cation transmembrane transporter activity	2.59	0.009
Solute cation antiporter activity	2.50	0.015
Ubiquitin like protein ligase activity	2.47	0.015

Top5 negatively enriched gene sets (WT-M/V, C5-MF)

Name	NES	FDR
Extracellular matrix structural constituent conferring tensile strength	–3.83	0.000
Extracellular matrix structural constituent	–3.65	0.000
Extracellular matrix structural constituent conferring compression resistance	–3.10	0.000
Aminopeptidase activity	–2.34	0.041
Intergin binding	–2.19	0.090

D

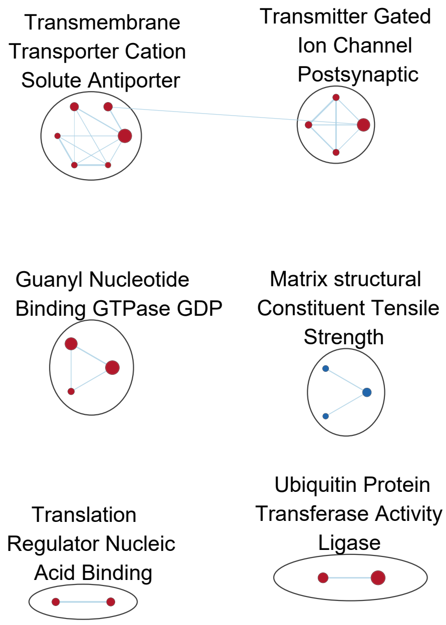

Supplementary Figure 7

A

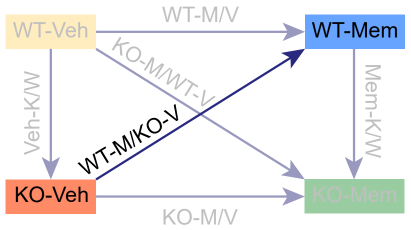

Top5 positively enriched gene sets (WT-M/KO-V, C5-BP)

Name	NES	FDR
Oxidative phosphorylation	5.58	0.000
ATP synthesis coupled electron transport	5.32	0.000
Respiratory electron transport chain	5.25	0.000
Electron transport chain	5.11	0.000
Cellular respiration	4.91	0.000

Top5 negatively enriched gene sets (WT-M/KO-V, C5-BP)

Name	NES	FDR
Defense response to virus	–3.04	0.004
Rhythmic process	–2.65	0.055
Circadian rhythm	–2.54	0.081
Telomere organization	–2.52	0.069
Response to virus	–2.42	0.118

B

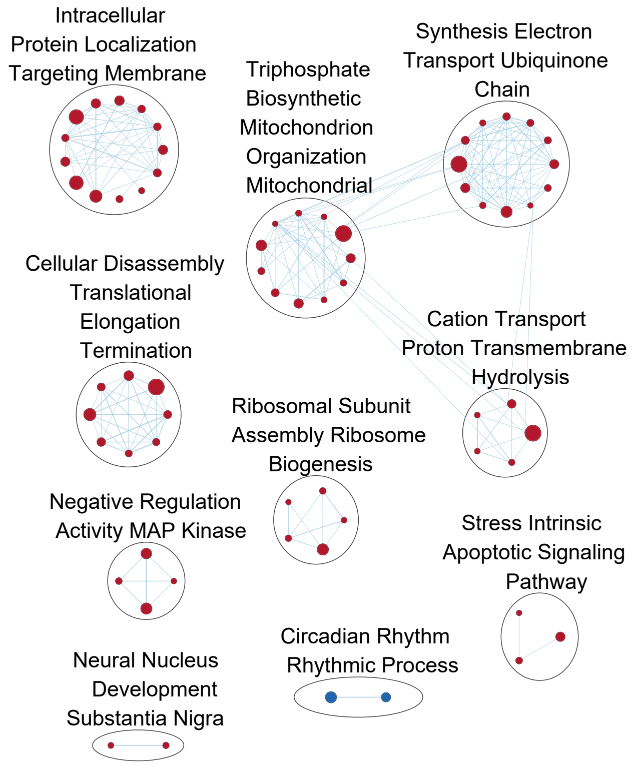

C

Top5 positively enriched gene sets (WT-M/KO-V, C5-MF)

Name	NES	FDR
Structural constituent of ribosome	5.79	0.000
NADH dehydrogenase activity	4.02	0.000
Electron transfer activity	3.90	0.000
Proton transmembrane transporter activity	3.57	0.000
Oxidoreductase activity acting on NADPH	3.49	0.000

Top5 negatively enriched gene sets (WT-M/KO-V, C5-MF)

Name	NES	FDR
Anion cation symporter activity	–2.56	0.046
Chloride transmembrane transporter activity	–2.48	0.040
Chromatin binding	–2.32	0.087
Peptide receptor activity	–2.28	0.083
Intergin binding	–2.25	0.080

D

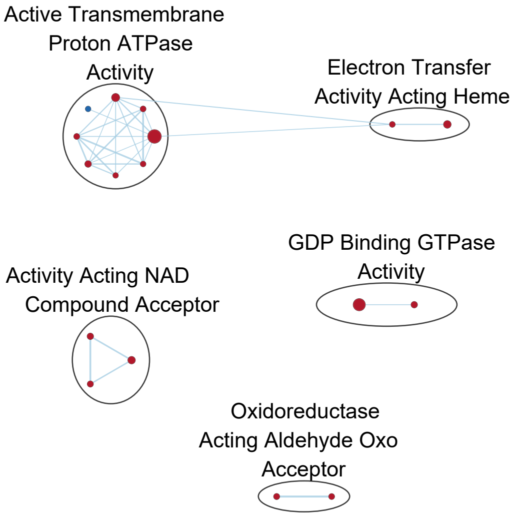

Supplementary Figure 8

A

			Veh-K/W		Mem-K/W		KO-M/V		WT-M/V	
Type	Gene set	Top1 gene	FC	Adjusted p-value	FC	Adjusted p-value	FC	Adjusted p-value	FC	Adjusted p-value
Veh-K/W	Postsynaptic membrane	HTR2A	1.35	2.04E-03	1.06	7.75E-01	-1.05	9.42E-01	1.21	1.00E+00
KO-M/V	Chromatin	MUC1	1.02	9.81E-01	1.85	5.41E-01	2.53	1.43E-01	1.38	1.00E+00
KO-M/V	U2 type spliceosomal complex	SF3B1	1.10	3.36E-01	1.27	7.99E-03	1.20	2.21E-01	1.04	1.00E+00

B

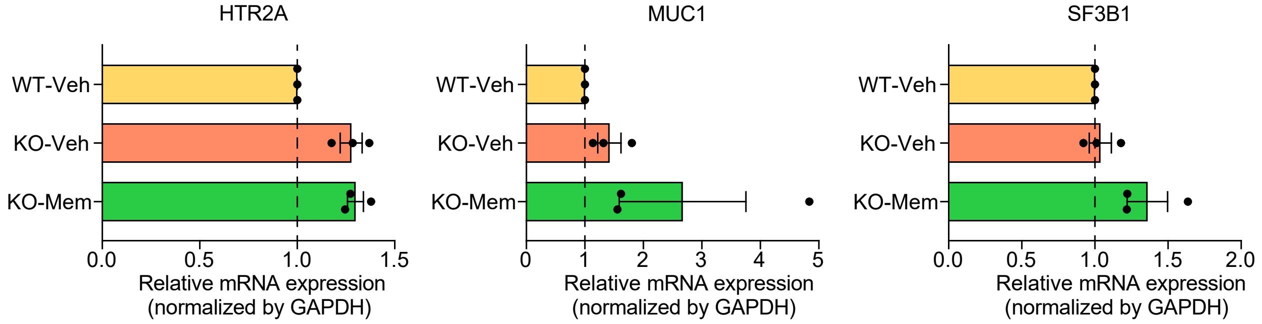

Supplementary Figure 9

A

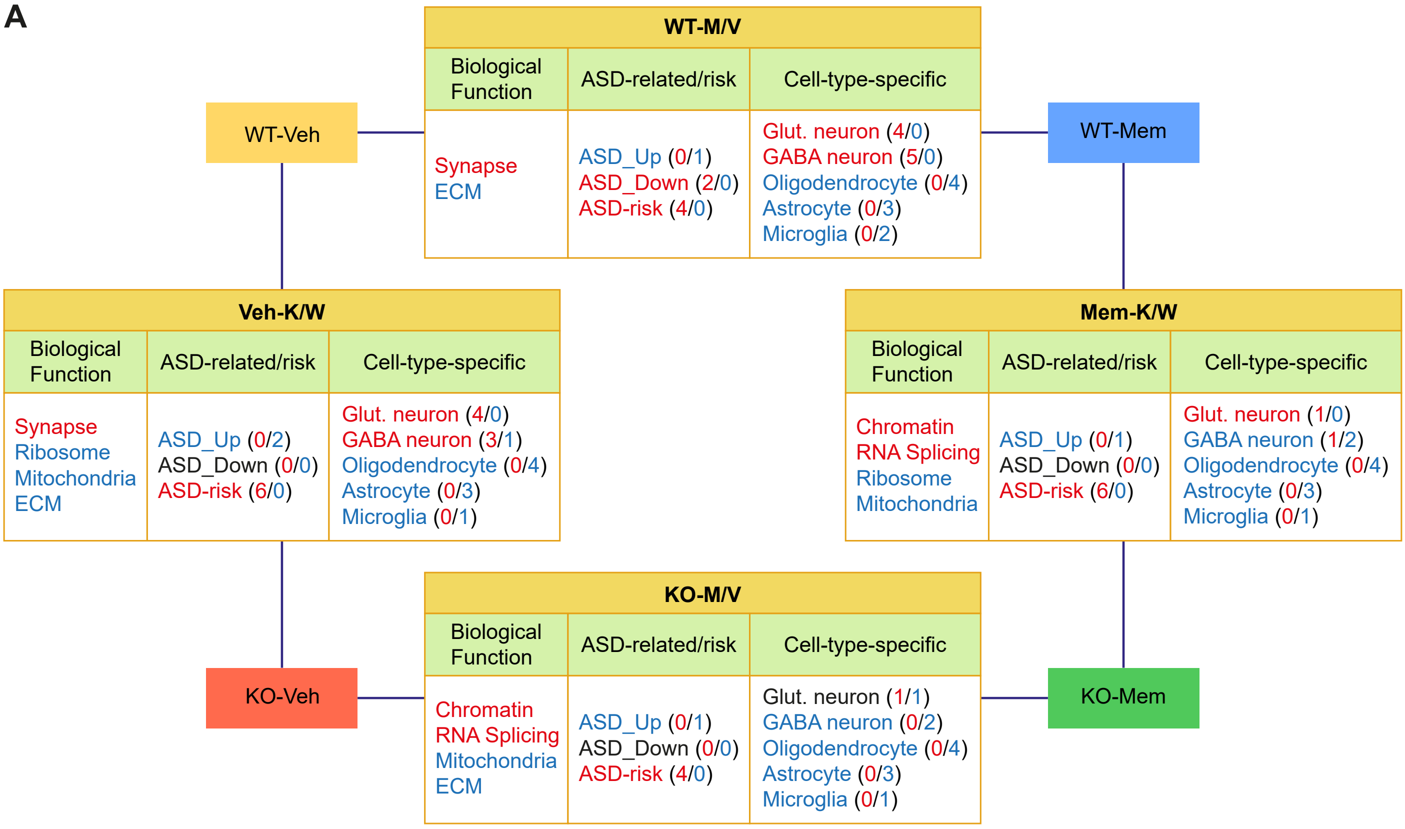

Supplementary Figure 10

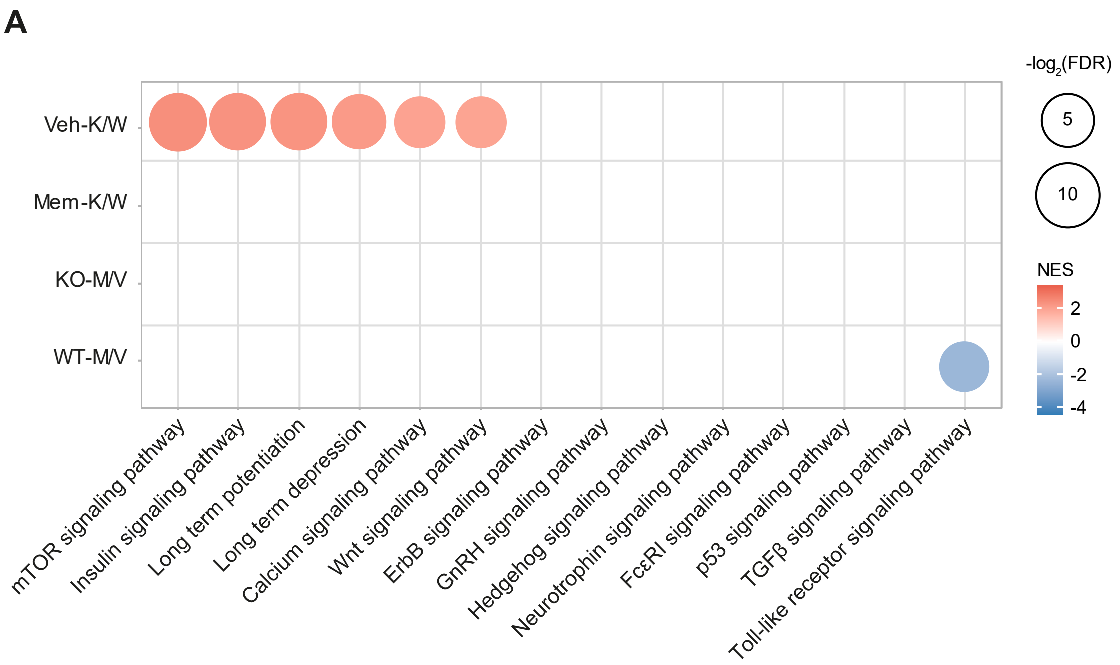

Supplement: Supplementary Figure 1 — Results of PCA analysis for the four RNA sample groups and statistical power analysis for sample size. (A) Principal component analysis (PCA) plots for WT-Veh, WT-Mem, KO-Veh, and KO-Mem transcriptomes. (B) Statistical power calculated for sample size of 3, 6, and 9 per group in each of the six transcript comparisons (Veh-K/W, Mem-K/W, KO-M/V, K-M/W-V, WT-M/V, and WT-M/KO-V). [file Data_Sheet_1.PDF]
